# Ultraplexing: increasing the efficiency of long-read sequencing for hybrid assembly with *k*-mer-based multiplexing

**DOI:** 10.1186/s13059-020-01974-9

**Published:** 2020-03-14

**Authors:** Alexander T. Dilthey, Sebastian A. Meyer, Achim J. Kaasch

**Affiliations:** 1 Institute of Medical Microbiology and Hospital Hygiene, University Hospital, Heinrich-Heine-University Düsseldorf, Düsseldorf, Germany; 2 Genome Informatics Section, Computational and Statistical Genomics Branch, National Human Genome Research Institute, Bethesda, MD 20892 USA; 3 Institute of Medical Microbiology and Hospital Hygiene, University Hospital, Otto-von-Guericke-University Magdeburg, Magdeburg, Germany

**Keywords:** Bacterial genomics, Genome assembly, Assembly graph, Multiplexing, *k*-mer, Hybrid assembly, Barcoding

## Abstract

Hybrid genome assembly has emerged as an important technique in bacterial genomics, but cost and labor requirements limit large-scale application. We present Ultraplexing, a method to improve per-sample sequencing cost and hands-on time of Nanopore sequencing for hybrid assembly by at least 50% compared to molecular barcoding while maintaining high assembly quality. Ultraplexing requires the availability of Illumina data and uses inter-sample genetic variability to assign reads to isolates, which obviates the need for molecular barcoding. Thus, Ultraplexing can enable significant sequencing and labor cost reductions in large-scale bacterial genome projects.

## Background

Accurate characterization of large numbers of microbial genomes is becoming increasingly important in microbiology. For example, bacterial genome-wide association studies (bGWAS) rely on the sequencing of large numbers of samples to correlate genetic variants to phenotypes such as antibiotic resistance or virulence [1–3]. Further examples are phylogenetic analyses and quality assurance in industrial microbiology [4–7].

A variety of sequencing technologies with different technological trade-offs have emerged for the sequencing of microbial genomes. Short-read sequencing technologies (such as Illumina [8] have low error rates (< 0.1%) but provide only limited resolution of complex and repetitive genomic regions. Examples are the genes encoding *S. aureus* protein A (*spa*) and fibronectin binding-protein (*fnbpA*), which play key roles in the pathogenesis of *S. aureus* [9] and which cannot be reliably assembled from short-read data [10]. Long-read sequencing technologies (Pacific Biosciences [11], Oxford Nanopore [12]) generate sequencing reads of tens or even hundreds of kilobases in length, enabling the correct structural resolution of complex regions; their higher error rates (5–15%), however, can negatively impact consensus and small-variant genotyping accuracy [13–15].

Combining short- and long-read data has therefore emerged as a standard approach for the resolution of bacterial genomes [16]. Long-read sequence information can be used to deconvolute short-read-based assembly graphs (hybrid de novo assembly [17–20]). Alternatively, de novo assemblies from long reads [21] can be polished with short-read data to improve consensus accuracy [22]. By either approach, the coverage requirements to arrive at a high-quality assembly of a microbial genome are typically modest (50–100× for each data type [23, 24]).

Molecular barcoding approaches enable the cost-effective sequencing of multiple samples in one run (“multiplexing”). Molecular barcoding involves the labeling of each DNA sample with a unique barcode sequence, pooling and joint sequencing of the samples, and determining the source sample for each sequencing read, based on its barcode sequences. Highly efficient, automated implementations of molecular barcoding exist for the Illumina platform, enabling the sequencing of hundreds of microbial isolates to sufficient coverage with a single flow cell. Molecular barcoding approaches for long-read platforms, however, are less effective. A maximum of 24 samples can currently be multiplexed on an Oxford Nanopore MinION flow cell using the manufacturer’s kits for “native” (PCR-free) barcoding. In addition, the preparation of multiplex libraries requires significant hands-on time (> 12 h compared to 3 h for a non-multiplexed library) and comes with significant losses of input material, and presumably, the pipetting steps reduce attainable read lengths by shearing. These factors make barcoded long-read sequencing costly and labor-intensive, and the availability of a more scalable approach to multiplexed long-read sequencing would be highly desirable.

**Fig. 1:**
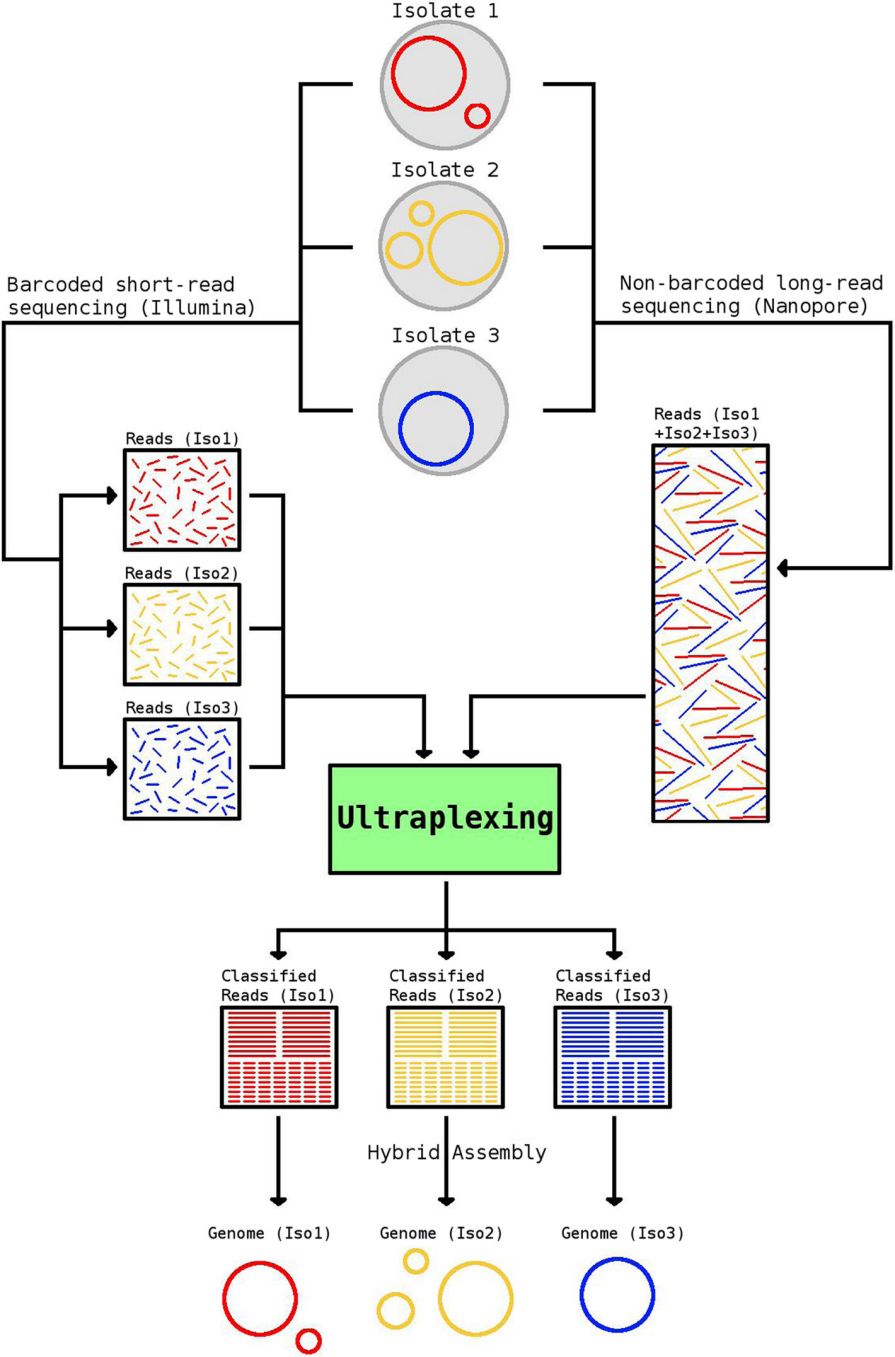
Overview of the Ultraplexing approach. Long reads are generated in simple pooled sequencing runs. The Ultraplexing algorithm determines the most likely source genome for each long read by carrying out a comparison between the read and the de Bruijn graphs of the sequenced sample genomes, inferred from short-read data. Hybrid assembly of sample-specific long and short reads enables the recovery of complete bacterial genomes

Here, we present Ultraplexing, a new method that allows the pooling of multiple samples in long-read sequencing without relying on molecular barcodes. Ultraplexing uses inter-sample genetic variability, as measured by Illumina sequencing, to assign long reads to individual isolates (Fig. 1). Specifically, each isolate genome is represented by its de Bruijn graph, constructed from sample-specific short-read data, and each long read is assigned to the sample de Bruijn graph it is most compatible with (or randomly in cases of a draw). A similar approach enables haplotype-aware assembly in eukaryotic genomes [25].

The intuition behind Ultraplexing is that there will typically be a high-quality alignment between a read and the assembly graph of the source genome it emanates from. Importantly, the assignment of reads completely contained in genomic regions shared among multiple samples (e.g., due to mobile genetic elements or inter-sample genetic homology) may remain ambiguous. This, however, will typically have no or only a small effect on the accuracy of the hybrid assembly process, for the affected reads will spell equally valid assembly graph traversals in all compatible samples.

Ultraplexing requires the availability of Illumina data. It is applicable to studies that either incorporate the generation of these from the beginning, or it can serve as a cost-effective method to generate additional long-read data for samples that have already been short-read sequenced. In the following, we demonstrate that Ultraplexing can match or even outperform classical molecular long-read barcoding approaches in terms of assembly quality while enabling significant reductions in cost and hands-on time.

## Results

We used simulated and real Nanopore and Illumina sequencing data to evaluate the performance of Ultraplexing in the context of bacterial hybrid de novo assembly. In all experiments, we relied on Unicycler as an established method for hybrid assembly [17]. We primarily focused on the quality of the generated assemblies, i.e., structural accuracy (number of contigs, reference recall, assembly precision) and consensus accuracy (single nucleotide polymorphisms; SNPs), measured against the utilized reference genomes (in simulations) or barcoding-based assemblies (for real data). To distinguish between Ultraplexing-mediated effects and intrinsic assembly complexity for the selected isolates, we reported assembly accuracy for random (in all experiments) and perfect (in simulations) assignment of long reads. Additionally, we assessed the proportion of correctly assigned reads. Of note, all simulation experiments were based on conservative assumptions (e.g., 5 Gb throughput per long-read flow cell; see the “Methods” section for further details), and no mis-assemblies were identified through visual inspection in any of the Ultraplexing-based sets.

### Simulation experiment I: Multi-species Ultraplexing

In a first step, we evaluated Ultraplexing on a sample of 10 different clinically important bacterial species (Additional file 1), covering a wide range of genome sizes (2.0–6.3 Mb), GC contents (32–60%), and between-species mash [26] distances (0.02–0.20; Additional file 2). The Ultraplexing algorithm assigned all but 2 of 477,890 simulated long reads to the correct bacterial isolate (close to 100% classification accuracy, Additional file 12: Figure S1). Ultraplexing-based assemblies were highly concordant (Additional file 12: Figure S1 and Additional file 3) with the underlying reference genomes, achieving near-perfect structural agreement (average reference recall and assembly precision > 99.999%) and low divergence (average number of SNPs against the reference genome, 57). Furthermore, assembly accuracy metrics for Ultraplexing and perfect read assignment were virtually identical (for example, an average of 57 SNPs for Ultraplexing compared to 56 SNPs for perfect assignment; Additional file 12: Figure S2). To assess how the performance of multi-species Ultraplexing was affected when combining more than one strain per species, we repeated the experiment for 5 clinically important species, each represented by 2 strains (Additional file 2) with mash distance < 0.01 (Additional file 2) [23]. Ultraplexing-based assemblies were virtually identical to assemblies based on perfect read assignment (for example, identical SNP count observed for 6/10 genomes) and of generally very high quality (Additional file 12: Figure S3 and Additional file 4), except for two *E. coli* genomes; in these, large repeat structures (Additional file 1) led two assembly fragmentation (> 100 contigs) for both Ultraplexing and perfect read assignment.

### Simulation experiment II: Single-species Ultraplexing with 10–50 isolates

**Fig. 2:**
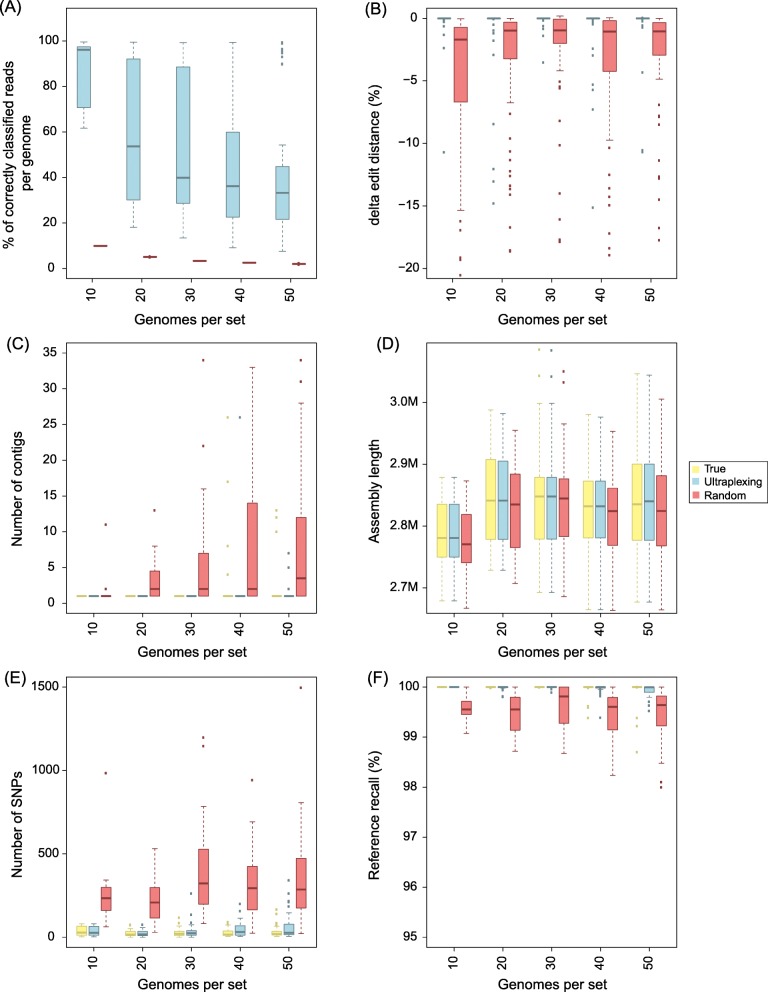
Simulated Ultraplexing runs with 10–50 *S. aureus* genomes, in comparison to perfect (True) and random (Random) assignment of long reads. **a** The proportion of correctly assigned long reads. **b** The ∆edit distance for random samples of falsely classified long reads. **c** The distribution of contigs per assembly. **d** The distribution of assembly lengths. **e** The distribution of SNPs per assembly. **f** The distribution of reference recall. SNPs and reference recall were calculated relative to the utilized reference genomes, and all metrics within the same set of genomes are based on the same simulated short-read data

To assess Ultraplexing performance on closely related isolates and with increasing sample numbers, we randomly selected sets of 10, 20, 30, 40, and 50 genomes from 181 publicly available complete assemblies of the human pathogen *Staphylococcus aureus* (Additional file 1). Of note, as simulated long-read flow cell capacity was held constant, sets with more genomes contained less long-read data per isolate. Across experiments, the proportion of correctly assigned reads decreased as sample numbers increased and varied between 35 and 95% (Fig. 2a). To test whether reduced read assignment accuracies were due to inter-sample sequence homologies, we computed the metric *∆edit distance* for random samples of mis-assigned reads and found an average *∆edit distance* of 0.3%, with more than 50% of mis-assigned reads exhibiting a *∆edit distance* of 0 (Fig. 2b). At the read alignment level, the genomes that the mis-assigned reads were assigned to are thus indistinguishable or very similar to the true source genomes. Consistent with this, the generated Ultraplexing-based assemblies were highly concordant with the utilized reference genomes (average reference recall ≥ 99.96% and assembly precision ≥ 99.99% across sets; average number of SNPs 46; Fig. 2c–f). Furthermore, assembly accuracy metrics for Ultraplexing and perfect read assignment were comparable even with increasing number of bacterial isolates; for example, the average number of SNPs per genome in the run with 50 bacterial isolates was 59 for Ultraplexing (QV 47) and 32 for perfect read assignment (QV 49). Complete results for this experiment are presented in Additional file 5 and visualized in Fig. 2. Finally, to evaluate to which extent assembly accuracy was influenced by genome complexity [23, 27], we repeated the experiment for 30 *S. aureus* isolates of class I complexity and for 30 *S. aureus* isolates of class III genome complexity (Additional file 1). Individual outliers in the set of class III genomes notwithstanding (Additional file 12: Figure S4), overall assembly quality remained high even for class III genomes (average reference recall, 99.98% for class compared to 99.86% for class III; average assembly precision, 100.00% for class I and III; average number of SNPs, 34 for class I and 77 for class III; Additional file 4). What is more, the quality of Ultraplexing-based assemblies remained comparable to that of assemblies based on perfect read assignment for class III genomes (for example, 77 SNPs on average for Ultraplexing, corresponding to QV 46, compared to 52 SNPs on average for perfect read assignment, corresponding to QV 47).

### Simulation experiment III: Impact of plasmids

In addition to the chromosomal genome, many bacterial cells harbor plasmids. Plasmids are extrachromosomal circular strings of DNA that are generally much smaller than the chromosomal DNA. Plasmids can vary in copy number within each cell, and they often exhibit complex and repetitive sequence structures. Since plasmid sequences could reduce the performance of the Ultraplexing algorithm, we repeated the previous simulation experiments with sets of 10–50 *S. aureus* genomes that all harbored plasmids (Additional file 1; Additional file 12: Figure S5). We found that the accuracy of chromosomal genome assemblies was not affected by the presence of plasmids. Additionally, the plasmid recovery rate was comparable to assemblies based on reads assigned to their true source; complete recovery was achieved in 135 of 150 total isolate genomes with Ultraplexing, and in 137 with perfect read assignments. Identified reasons for incompletely recovered plasmids included high sequence homology to other plasmids or the genomic DNA (Additional file 6). Complete results for this experiment are presented in Additional file 7 and visualized in Additional file 12: Figure S6 (chromosomal genome) and Additional file 12: Figure S7 (plasmids). Finally, we further explored the impact of repeats between the chromosomal and plasmid genomes on a set of 10 complex (class III) *Pseudomonas* isolates, 9 of which harbor chromosome-plasmid repeats ranging from 669 bp to 69 kb in size (Additional file 1; Additional file 12: Figure S8). Assembly accuracy remained high at slightly reduced levels (reference recall > 97% and assembly precision > 99% for all 10 genomes), and Ultraplexing- and truth-based assemblies are almost identical in terms of accuracy metrics (identical reference recall for 10/10 isolates and identical assembly precision for 9/10 at very similar SNP levels; Additional file 4).

### Real-data experiment I: Nanopore-based Ultraplexing of 10 *S. aureus* clinical samples

To assess the performance of Ultraplexing on real data, we randomly selected ten bacterial isolates of the species *Staphylococcus aureus* from our collection of clinical isolates. To generate a reference genome for each isolate, we sequenced each sample on an Illumina system, performed barcoded Oxford Nanopore sequencing with the 12-sample barcoding kit (~ 214× coverage per isolate; mean read length 8.3 kb), and carried out hybrid de novo assembly. The generated reference genomes consist of 1–3 circular contigs per isolate, representing the chromosomal genome (~ 2.8 Mb in length) and plasmids (2.3–34.9 kb in length, all circular; BLAST [28] classification results are shown in Additional file 8).

**Fig. 3:**
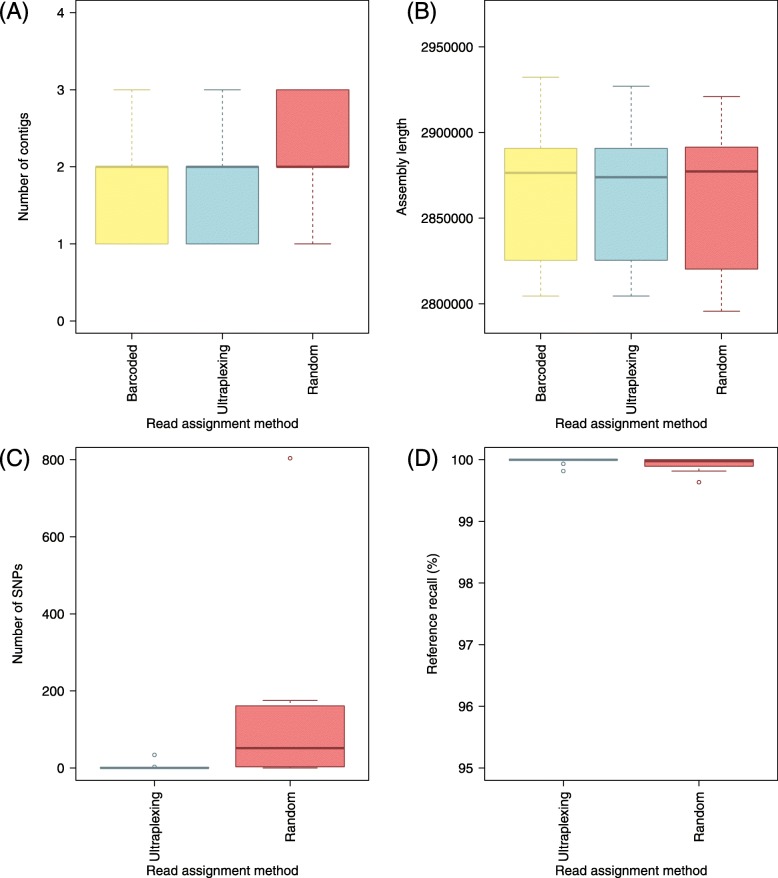
Ultraplexing and classical molecular barcoding on a set of ten *S. aureus* isolates. For different read assignment methods applied to the same set of Nanopore reads, the distribution of contigs per assembly (**a**), the distribution of assembly lengths (**b**), the distribution of SNPs per assembly (**c**), and the distribution of reference recall (**d**) are shown. SNPs and reference recall were calculated relative to assemblies based on molecular barcoding, and the same Illumina sequencing data were used throughout. Barcoded, reads assigned according to molecular barcodes; Ultraplexing, reads assigned by the Ultraplexing algorithm; Random, reads assigned randomly

To test Ultraplexing on these isolates, we demultiplexed the barcoded Nanopore sequencing data with the Ultraplexing algorithm and carried out hybrid de novo assembly. The Ultraplexing-based assemblies showed a high degree of concordance (Fig. 3) with the generated reference genomes in terms of contig number, assembly length, genome structure (average reference recall and assembly precision > 99.9%), and consensus accuracy (4 SNPs per isolate on average and 6 of 10 isolates with no detected SNPs). In contrast, assemblies based on random read assignment yielded lower-quality assemblies across all considered metrics (for example, 136 SNPs per genome; Fig. 3d). Complete results for all genomes are presented in Additional file 9 and visualized in Fig. 3. Summary statistics of the Illumina and Nanopore sequencing runs can be found in Additional file 10.

### Read-data experiment II: Nanopore-based Ultraplexing of 48 clinical isolates

To assess the feasibility of applying Ultraplexing to a larger number of samples, we repeated the previous experiment with 48 samples. As in the previous experiment, barcoded Nanopore (~ 446× coverage per isolate; average read length 10.4 kb) and Illumina (~ 44× coverage per isolate; 2 × 250 bp reads with MiSeq v2 chemistry) sequencing was carried out to generate reference genomes for the 48 samples.

For Ultraplexing, long-read sequencing data (~ 87× coverage per isolate; average read length 11.7 kb) was generated in a single MinION run by pooling DNA from the 48 isolates. Reads were demultiplexed with the Ultraplexing algorithm, and hybrid de novo assembly was carried out.

**Fig. 4:**
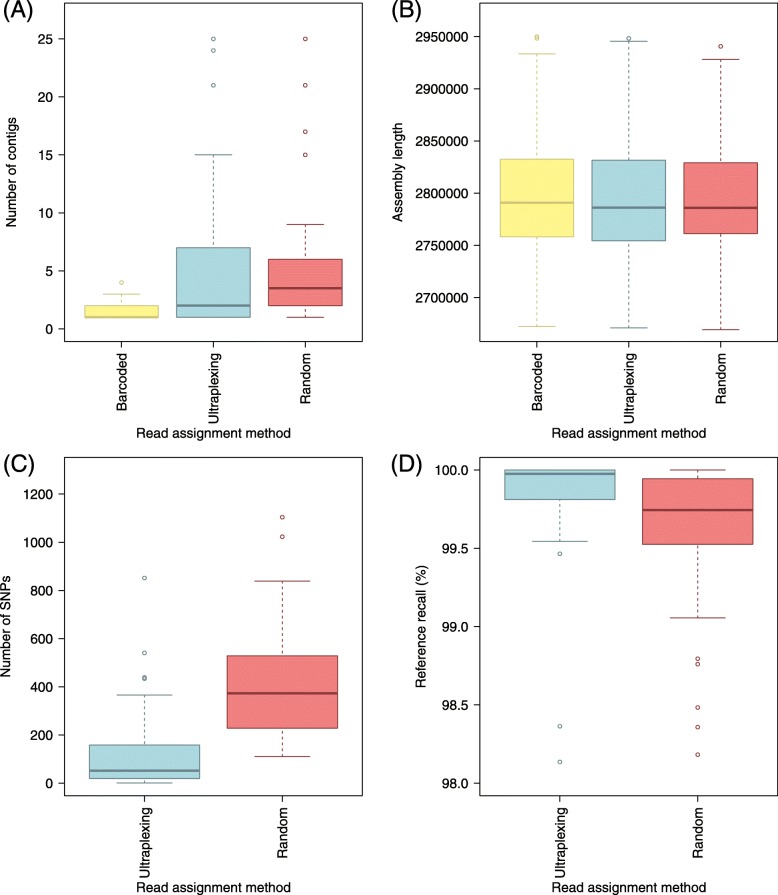
Ultraplexing and classical molecular barcoding on a set of 48 *S. aureus* isolates. **a** The distribution of contigs per assembly. **b** The distribution of assembly lengths. **c** The distribution of SNPs per assembly. **d** The distribution of reference recall. SNPs and reference recall are calculated relative to assemblies based on molecular barcoding, and the same Illumina sequencing data were used throughout. Barcoded, molecularly barcoded Nanopore data, 5 flow cells with ≤ 10 samples each; Ultraplexing, reads assigned by the Ultraplexing algorithm, 1 flow cell with 48 samples; Random, reads from the Ultraplexing run, assigned randomly

The generated assemblies exhibited a plausible profile in terms of assembly length, and for 29/48 assemblies, the Ultraplexing-based assembly had the same number of contigs as the generated reference genomes (Fig. 4). Further investigation showed a high degree of concordance between the Ultraplexing-based assemblies and the reference genomes both in terms of genome structure (average reference recall and assembly precision > 99.8%) and the number of SNPs per genome (126 on average, equivalent to QV 43). Complete results for the comparison of the 48 Ultraplexing-based assemblies against the reference genomes are presented in Additional file 9 and visualized in Fig. 4. Read length and coverage statistics for all sequencing runs can be found in Additional file 10; the read length distribution of all generated Nanopore sequencing runs is visualized in Additional file 12: Figure S9.

## Discussion

We have presented Ultraplexing, a method that resolves pooled long-read sequencing data in the context of hybrid de novo assembly without the use of barcoding. Ultraplexing leverages inter-sample genetic variation to assign pooled long reads to individual isolates and benefits from the fact that Illumina sequencing enables the reliable characterization of the *k*-mer spectra of individual genomes.

Using simulated sequencing data, we demonstrated that Ultraplexing enables the generation of highly accurate hybrid assemblies and reliably detects plasmids, even in datasets that contain multiple isolates of the same bacterial species, complex plasmid-chromosome repeat structures, or genomes of high complexity. We have also validated the method on two real Nanopore sequencing datasets and shown that Ultraplexing-based assemblies are virtually identical to barcoding-based assemblies when comparing multiplexed runs with the same number of isolates; remaining errors in the assemblies based on both Ultraplexing and perfect read assignment may represent residual errors introduced by the hybrid assembly approach. When using Ultraplexing to increase the number of samples over the current maximum of PCR-free molecular barcoding approaches on the Nanopore platform, Ultraplexing-based assemblies generally maintain high accuracy.

A key advantage of Ultraplexing in comparison to molecular barcoding is decreased cost and hands-on time. The number of samples sequenced per flow cell can at least be doubled, and barcoding reagents are not necessary. Hands-on time was reduced eightfold in our 48-sample experiment (~ 5 h per flow cell with 10 barcoded samples compared to 3 h for one Ultraplexing run with 48 samples). Taking into account potential differences in sample handling operator performance, we conservatively estimate that the hands-on time benefit conferred by Ultraplexing is at least 50%.

On the other hand, Ultraplexing has a number of limitations. First, Ultraplexing can consume significant computational resources (70 CPU hours and 175 Gb of memory for the demultiplexing step in the experiment with 48 samples). Improvements in hands-on time do therefore not necessarily translate into decreased time-to-result. Second, Ultraplexing relies on Illumina data for read assignment and hybrid assembly; systematic biases in Illumina sequencing, as observed for certain bacterial genomes with high or low GC content [29], may affect the accuracy of Ultraplexing. Third, the application of Ultraplexing requires high molecular weight DNA, the extraction of which may be challenging for certain bacterial species. Fourth, while we have shown that Ultraplexing is generally robust against the presence of complex repeat structures, assembly accuracy was slightly reduced for class III genomes. For these reasons, the method is best suited to applications in which large numbers of genomes need to be resolved to very high, but not perfect, accuracy, and in which turnaround times on the order of 3–5 days are acceptable. Examples of this include bacterial genome-wide association studies and retrospective outbreak sequencing. For other applications, such as the generation of a small number of reference-grade assemblies or time-critical diagnostic applications, conventional barcoding approaches may remain preferable.

Although our primary focus was on assembly accuracy, we also evaluated the accuracy of individual read assignments in the simulation experiments. One important factor driving read assignment accuracy was the extent of genetic variability between the pooled samples. Consistent with this, Ultraplexing achieved near-perfect read assignment in the first multi-species experiment but reduced assignment accuracy when species were represented by more than one strain. We hypothesized that mis-assignments driven by inter-sample sequence homology would have a negligible effect on assembly accuracy. Consistent with this, assembly accuracy was relatively insensitive to increasing numbers of mis-assigned reads in the single-species experiment, and we could confirm that inter-sample sequence homology accounts for the majority of mis-assigned reads using edit distance metrics. Furthermore, assembly accuracy was significantly reduced for random read assignment, reflecting higher proportions of falsely assigned reads in the absence of underlying sequence homologies. In addition, Ultraplexing may be less well suited for applications that depend on accurate assignments of individual reads, such as read-based methylation calling.

Our study has a number of limitations. First, we have only validated Ultraplexing on a single long-read technology, Oxford Nanopore. However, based on prior work demonstrating successful *k*-mer-based classification of eukaryotic PacBio reads [30, 31], we expect that Ultraplexing could also be applied to PacBio data, though the shorter subread distribution of the technology may negatively impact accuracy [32]. Second, although Ultraplexing was validated on a number of clinically important bacterial species covering a wide array of genome sizes and genome complexities, we cannot exclude the possibility that performance may degrade for genome or repeat configurations not included in the test set. Third, we have not rigorously tested the technical limits of Ultraplexing, including the maximum number of isolates and the necessary properties of the short-read sequencing data. Given that flow cell output has been increasing steadily, extraction of high molecular weight DNA for long-read sequencing may plausibly become the most significant limiting factor. Fourth, in terms of bioinformatics methods development, Ultraplexing relies on simple *k*-mer statistics instead of proper graph alignment [33–35], and we have not explored methods for the optimization of intra-batch genetic diversity in large sequencing projects. These points could be addressed in future work.

## Conclusion

Ultraplexing is a new method for multiplexed long-read sequencing in the context of hybrid de novo assembly. Ultraplexing-based assemblies are highly accurate in terms of genome structure and consensus accuracy and exhibit quality characteristics comparable to assemblies based on molecular barcoding. Through increasing the number of samples per flow cell and simplified library preparation, Ultraplexing enables significant reductions of long-read sequencing costs and hands-on time. Thus, Ultraplexing enables the cost-effective complete resolution of large numbers of bacterial genomes.

## Methods

### The Ultraplexing read assignment algorithm

Let *n* denote the number of sequenced bacterial samples. We assume the availability of high-coverage Illumina sequencing data for each of the *n* individual isolates and that a pool of high molecular weight DNA, representing a mixture of the genomes of the *n* isolates, has been sequenced with a long-read sequencing technology like Oxford Nanopore or Pacific Biosciences. For each sample, a de Bruijn graph (*k* = 19) is constructed from the sample-specific Illumina short-read data and the graph is cleaned (removal of low-coverage supernodes) with Cortex [16]. Each long read from the pooled run is assigned to the sample for which the number of read *k*-mers present in the cleaned sample de Bruijn graph is maximal (or randomly in cases of a draw). We note that our approach can be understood as a heuristic approach to read-to-graph alignment. After the long-read assignment process is complete (i.e., after each long read has been assigned to one of the *n* isolates), the Cortex graph is discarded for the subsequent assembly steps. Of note, the choice of a *k* is a trade-off between the number of isolate-specific *k*-mers at a given *k* and the expected *k*-mer survival rate in the long-read data, calculated as (1 − *e*)^*k*, where *e* is the long-read sequencing error rate. *k* = 19 was chosen based on published work [25] on *k*-mer-based binning of long reads and based on preliminary simulation experiments.

### Hybrid assembly and assembly evaluation criteria

Unicycler (version 0.4.4) [17] was used for all hybrid assembly experiments in this publication. Unicycler receives, for each sample, (I) the sample-specific Illumina reads and (II) the long reads assigned to the sample. Long reads are assigned according to the Ultraplexing long-read assignment algorithm, the molecular barcodes, or the underlying ground truth, depending on the evaluation scenario.

The performance of Ultraplexing was assessed (I) by assessing the proportion of reads assigned to the correct sample (in simulations), (II) by comparing the generated Ultraplexing-based hybrid de novo assemblies to reference genomes (downloaded from RefSeq for simulations and based on barcoding-based hybrid assembly for real data, see below), and (III) by comparing the accuracy of Ultraplexing-based assemblies to that of assemblies based on random (all experiments) or perfect (in simulations) assignment of long reads.

To assess the accuracy of an assembly, we compared the assembly to the corresponding reference genome. As baseline characteristics, we considered the total number of contigs and the combined assembly length. Furthermore, nucmer v3.1 [36] was used to generate an alignment between the assembly and the reference genome, globally filtering identified diagonals with “delta-filter -1.” We used the filtered diagonals to compute three quality metrics: “SNPs,” measuring consensus accuracy; “reference recall,” the fraction of the reference covered by the assembly; and “assembly precision,” the fraction of the assembly covered by the reference. When reported, QV scores are calculated as $round\left(-10\times \log 10\left(\frac{average\# SNPs\kern0.2em per\kern0.2em genome}{ average\ reference\ genome\ size}\right)\right)$ (Phred scale). Of note, assembly precision was close to 100% in all experiments, and we do not separately report on this metric.

For the simulation experiment with plasmids, we separately evaluated the sets of chromosomal and plasmid contigs for each assembly. We relied on RefSeq annotations for determining the status (chromosomal or plasmid) of each contig in the reference and assigned the status of each assembly contig according to the status of its highest-scoring nucmer hit in the reference.

### Read assignment accuracy and edit distance

In simulated datasets, we calculated the proportion of correctly assigned long reads. A read was counted as correctly assigned if, and only if, it was assigned to the genome it was simulated from. For mis-assigned reads, we additionally defined a metric referred to as “∆edit distance,” using edlib (version 1.2.6) [37]. Let *d*_1_ be the ends-free edit distance between a read and the genome it was simulated from, and let *d*_2_ be the edit distance between a read and the genome it was assigned to. ∆edit distance is defined as *d*_1_–*d*_2_, divided by the length of the read. A negative value indicates a better alignment to the source genome than to the predicted genome. To assess the distributional properties of ∆edit distance, the metric was calculated for random samples of 100 mis-assigned reads per method.

### Simulation experiments

For the multi-species simulation experiments, chromosomal sequences of 10 clinically important species were downloaded from RefSeq [38]. For the single-species experiments without plasmids, chromosomal sequences of 181 complete *S. aureus* genomes were downloaded from RefSeq [38]. For the single-species simulation experiment with plasmids, 169 complete genomes were downloaded that contained between 2 and 11 annotated plasmids. The accessions of all downloaded genomes are listed in Additional file 1, and the selected genome subsets are listed in the corresponding results tables (Additional files 4 and 5).

For each genome, 300 Mb of short-read data was simulated with wgsim (version 0.3.1-r13) [39], using the parameters base error rate (-e 0.005), length of first read (-1 150), length of second read (-2 150), outer distance between the read ends (-d 278), standard deviation (-s 128), mutation rate (-r 0), and fraction of indels (-R 0). Long-read data were simulated with pbsim (version 1.0.3 )[40], using the parameters prefix of the output (--prefix [prefix]), coverage (--depth 200), mean read length (--length-mean 8370), standard deviation of the read length (--length-sd 6389), maximum read length (--length-max 61011), minimum read length (--length-min 230), mean sequencing accuracy (--accuracy-mean 0.88), and model of quality code (--model_qc model_qc_clr). Mean read length was adjusted to match that of our first Nanopore sequencing run, and maximum read length was set to approximately 85% of that observed on the first run (Additional file 10). For all experiments, we assumed a constant long-read flow cell capacity of 5 Gb, and per-isolate coverage was adjusted accordingly (i.e., 5 Gb total output divided by the number of simulated isolates). Simulated long-read data were pooled and demultiplexed with the Ultraplexing algorithm. Hybrid de novo assembly was carried out, and the generated assemblies were benchmarked against the utilized reference genomes.

### DNA extraction and long-read sequencing

DNA was extracted from overnight bacterial cultures in 3 ml LB broth. For short-read sequencing, the “DNeasy UltraClean Microbial” Kit was used according to the manufacturer’s instruction. One nanogram of DNA per isolate was used for the library preparation with the TruePrep DNA Library Prep Kit. Short-read sequencing was conducted on a MiSeq instrument (Illumina) using 250 bp paired end sequencing using v2 chemistry. DNA extraction for long-read sequencing was performed with the MagAttract HMW DNA Kit (QIAGEN). Wide bore pipette tips were used to avoid shearing. Long-read sequencing was carried out on a MinION device with FLO-MIN106 flow cells and the SQK-LSK108 ligation sequencing kit (real-data experiment I) and SQK-LSK109 ligation sequencing kit (real-data experiment II). Of note, SQK-LSK109 involves reduced pipetting, possibly decreasing shearing. For barcoded long-read sequencing, samples were labeled with barcodes using the Oxford Nanopore ligation sequencing kit (EXP-NBD103 kit for 12 samples per run), and reads were demultiplexed with Albacore (version 2.1.3). For Ultraplexing, DNA from individual samples was pooled based on equal weight to yield a total of 700 ng of DNA, and demultiplexing was carried out with the Ultraplexing algorithm. Summary statistics of all sequencing runs are presented in Additional file 10.

### Real-data validation experiments

For all experiments with real data, we used hybrid assembly with Unicycler [17] to generate high-quality reference genomes for all isolates, combining molecularly barcoded short- and long-read data.

Molecular long-read barcoding was carried out using the 12-sample barcoding kit (EXP-NBD103) for the first real-data experiment (1 flow cell) and for the second real-data experiment (5 flow cells with ≤ 10 samples per run). Barcoded Illumina sequencing runs were carried out for all samples in the real-data experiments. All sequencing runs are summarized in Additional file 10. Read mappability was determined with BWA MEM (version 0.7.17-r1188) (with standard settings and read mapping mode -x ont2d) [41].

### Plasmid identification

To check if smaller contigs in barcoded assemblies of the real-data experiments represented plasmids, we used the online version of BLAST [28]. All non-chromosomal contigs (assumed to be all contigs but the longest in each assembly) were blasted against the nucleotide (nt) database, restricted to sequences that correspond to bacteria (taxid:2), and if the best hit was characterized as plasmid and had a high identity (≥ 90%) and a low *e* value (0 or close to 0), we assumed that the contig represented a correctly assembled plasmid (Additional file 8). Three plasmids that generated hits to human BAC constructs were removed from the corresponding assemblies.

## Supplementary information

**Additional file 1.** Sample summary. Names, accessions and summary statistics of all utilized reference genomes.

**Additional file 2.** Mash distances. Relatedness of genomes within each experiment.

**Additional file 3.** Main Evaluation Simulation Experiment I. Read classification and assembly accuracy in a simulation experiment with 10 different human pathogens.

**Additional file 4. **Evaluation 3 Additional Simulation Experiments. Read classification and assembly accuracy for 3 additional simulation experiments (5 species x 2 strains, class I and class III *S. aureus*, 10 *Pseudomonas*).

**Additional file 5. **Main Evaluation Simulation Experiment II. Read classification and assembly accuracy in a simulation experiment with 10 – 50 *S. aureus* genomes.

**Additional file 6. **Incorrectly assembled plasmids (simulations). Incorrectly assembled or incompletely recovered plasmids in the simulated sets with 10 – 50 plasmid-containing *S. aureus* isolates.

**Additional file 7. **Main Evaluation Simulation Experiment III. Read classification and assembly accuracy in a simulation experiment with 10 – 50 plasmid-containing *S. aureus* genomes.

**Additional file 8.** Putative plasmids (real data). BLAST results for contigs putatively representing plasmids in two real-data experiments.

**Additional file 9.** Evaluation of real-data experiments. Assembly accuracy and properties of the utilized reference genomes in two real-data experiments.

**Additional file 10.** Sequencing data summary. Summary statistics of all generated read sets (Oxford Nanopore and Illumina).

**Additional file 11.** Detailed legends for the supplementary tables.

**Additional file 12.** Supplementary figures.

**Additional file 13.** Review history.

## Data Availability

The datasets generated and analyzed during the current study, as well as the generated reference assemblies, are available under the BioProject accession number PRJNA528186: https://www.ncbi.nlm.nih.gov/bioproject/PRJNA528186 [42]. Assemblies from Ultraplexing-based and random assignment of reads and the source code of the Ultraplexer are available on OSF: 10.17605/OSF.IO/4M9VH [43]. The source code of the Ultraplexing algorithm is also available on GitHub: https://github.com/SebastianMeyer1989/UltraPlexer [44]. The Ultraplexing algorithm is made available under the MIT license and implemented in C++, Perl, and R. Sequence-to-graph alignment depends on the Cortex (cortex_var) package version 1.0.5.21 [16].
