# Supplementary material for: Ultraplexing: increasing the efficiency of long-read sequencing for hybrid assembly with k-mer-based multiplexing
Source: Genome Biol. 2020 Mar 14;21:68. doi: 10.1186/s13059-020-01974-9 (PMC7071681; doi:10.1186/s13059-020-01974-9)
Supplement: Supplementary file 11 — Additional file 11. Detailed legends for the supplementary tables. [file 13059_2020_1974_MOESM11_ESM.pdf]

Table S1:
Names, NCBI accessions, complexity, repeat richness, and GC content of the genomes underlying the simulation experiment with ten different human pathogens (Tab1: Multi-species_10); the simulation experiments with 10 – 50 *S. aureus* genomes (Tab2: Single-species_10-50); the simulation experiments with 10 – 50 plasmid-containing *S. aureus* genomes (Tab3: Single-species_10-50 plus plasmids); the simulation experiment with five different human pathogens, each represented by two strains (Tab4: Multi-species_5x2); the simulation experiment with two sets of 30 S. aureus genomes with different genome complexities (I and III) (Tab5: Single-species_high_complexity); the simulation experiment with 10 Pseudomonas genomes with high repeat richness between genomic sequences and plasmids (Tab6: Single-species_repeat_richness); and name, complexity, repeat richness and GC content of the genome assemblies created from real sequencing data (Tab7: Real Data). The second and third sheet specify the sets of genomes of which the 10 – 50 samples in simulation experiments II and III were drawn from. Note that "Calculated complexity" specifies genomic complexity classes as defined by Koren et al. (2013), whereas the values in "Published complexity" are reproduced from Schmid et al. (2018), where available.

Table S2:
Relatedness of genomes used within each experiment, represented in a matrix of mash distances, with values ranging from 0 ("identical") and 1 ("unrelated"). Shown are mash distances for the simulation experiment with ten different human pathogens (Tab1: Multi-species_10); the simulation experiments with 10 – 50 *S. aureus* genomes (Tab2: Single-species_10-50); the simulation experiments with 10 – 50 plasmid-containing *S. aureus* genomes (Tab3: Single-species_10-50 plus plasmids); the simulation experiment with five different human pathogens, each represented by two strains (Tab4: Multi-species_5x2); the simulation experiment with two sets of 30 S. aureus genomes with different genome complexities (I and III) (Tab5: Single-species_high_complexity); the simulation experiment with 10 Pseudomonas genomes with high repeat richness between genomic sequences and plasmids (Tab6: Single-species_repeat_richness); and the genome assemblies created from real sequencing data (Tab7: Real Data).

Table S3:
Read classification and assembly accuracy in a simulation experiment with ten different human pathogens. The first sheet shows the number and proportion and correctly classified reads for Ultraplexing and random read assignment (Tab 1: Classifier); the second sheet shows Δedit distance data for a subsample of individual reads falsely classified by the 'random' assignment method (Tab 2: Prediction Score); the third sheet shows assembly metrics (contig lengths, assembly precision, reference recall, SNPs and N50/L50) for different long-read assignment methods (Tab 3: Assembly). Metrics with the suffix '_longest' refer to the longest contig in the corresponding assembly. 'True': long reads assigned to their true origin; 'predicted': long reads assigned by the Ultraplexing algorithm; 'random': long reads assigned randomly. Individual columns are visualized in Supplementary Figures 1 and 2.

Table S4:
Read classification and assembly accuracy for all additional simulations. The first sheet shows the number and proportion and correctly classified reads for Ultraplexing and random read assignment (Tab 1: Classifier); the second sheet shows Δedit distance data for a subsample of individual reads falsely classified(Tab 2: Prediction Score); the third to fifth sheet shows assembly metrics (contig lengths, assembly precision, reference recall, SNPs and N50/L50) for different long-read assignment methods (Tab 3-5: Assembly Sim1-Sim3). Metrics with the suffix '_longest' refer to the longest contig in the corresponding assembly. 'True': long reads assigned to their true

origin; ‘predicted’: long reads assigned by the Ultraplexing algorithm; ‘random’: long reads assigned randomly. Individual columns are visualized in Supplementary Figures 3, 4 and 8.

Table S5:
Read classification and assembly accuracy in five simulation experiments with 10 – 50 different *S. aureus* genomes. The first sheet shows the number and proportion and correctly classified reads for Ultraplexing and random read assignment (Tab 1: Classifier); the second sheet shows Δedit distance data for subsamples of individual reads falsely classified (Tab 2: Prediction Score); the third sheet shows assembly metrics (contig lengths, assembly precision, reference recall, SNPs and N50/L50) for different long-read assignment methods (Tab 3: Assembly). Metrics with the suffix ‘_longest’ refer to the longest contig in the corresponding assembly. ‘True’: long reads assigned to their true origin; ‘predicted’: long reads assigned by the Ultraplexing algorithm; ‘random’: long reads assigned randomly. Individual columns are visualized in Figure 2.

Table S6:
Incorrectly assembled or incompletely recovered plasmids in the simulated sets with 10 – 50 plasmid-containing *S. aureus* isolates. The table shows the difference in contig number between the assembly and the source genome and a description of the identified assembly error.

Table S7:
Read classification and assembly accuracy in five simulation experiments with 10 – 50 different plasmid-containing *S. aureus* genomes. The first sheet shows the number and proportion and correctly classified reads for Ultraplexing and random read assignment (Tab 1: Classifier); the second sheet shows Δedit distance data for subsamples of individual reads falsely classified (Tab 2: Prediction Score); the third sheet shows assembly metrics (contig lengths, assembly precision, reference recall, SNPs and N50/L50) for different long-read assignment methods (Tab 3: Assembly). For assembly accuracy metrics, reference and assembly contigs were classified as ‘chromosomal’ or ‘plasmid’ (see Methods) and evaluated separately (column ‘Type_2’). Metrics with the suffix ‘_longest’ refer to the longest contig in the corresponding assembly. ‘True’: long reads assigned to their true origin; ‘predicted’: long reads assigned by the Ultraplexing algorithm; ‘random’: long reads assigned randomly. Individual columns are visualized in Supplementary Figures 5, 6 and 7.

Table S8:
BLAST results for contigs putatively representing plasmids in two real-data experiments, based on assemblies of molecularly barcoded data. The table shows identity and E-value of the best BLAST hit for each contig. The first sheet (Tab 1: Ultraplex_10_Isolates) shows results for the first real-data experiment (sequencing of 10 *S. aureus* isolates) and the second sheet (Tab 2: Ultraplex_48_Isolates) shows results for the second real-data experiment (sequencing of 48 *S. aureus* isolates). The best hit (corresponding to identity and e-value) for each possible plasmid is shown.

Table S9:
Assembly accuracy in real-data experiments. The first sheet (Tab 1: Assembly) shows results for a multiplexed sequencing run of 10 *S. aureus* isolates (Type_2: ultraplex_10x); and results for a multiplexed sequencing run of 48 different *S. aureus* isolates benchmarked against an initial set of reference genomes (Type_2: ultraplex_48x). ‘Predicted’: long reads assigned by the Ultraplexing algorithm; ‘random’: long reads assigned randomly. The second sheet (Tab 2: Reference) shows the properties of the utilized reference genomes

Table S10:
Summary statistics of all generated read sets (Oxford Nanopore and Illumina). The metrics were calculated for all reads of the corresponding data set and for the subset of mappable reads, determined with BWA. The data are shown in separate blocks each real-data experiment (sequencing of 10 isolates, sequencing of 48 isolates). The summary statistics of one simulated dataset (SIM_pool_10_nanopore.fastq) are shown for comparison; the same parameters were used for all simulations.
