# Supplementary material for: Ultraplexing: increasing the efficiency of long-read sequencing for hybrid assembly with k-mer-based multiplexing
Source: Genome Biol. 2020 Mar 14;21:68. doi: 10.1186/s13059-020-01974-9 (PMC7071681; doi:10.1186/s13059-020-01974-9)
Supplement: Supplementary file 12 — Additional file 12. Supplementary figures. [file 13059_2020_1974_MOESM12_ESM.pdf]

Supplementary Figures:

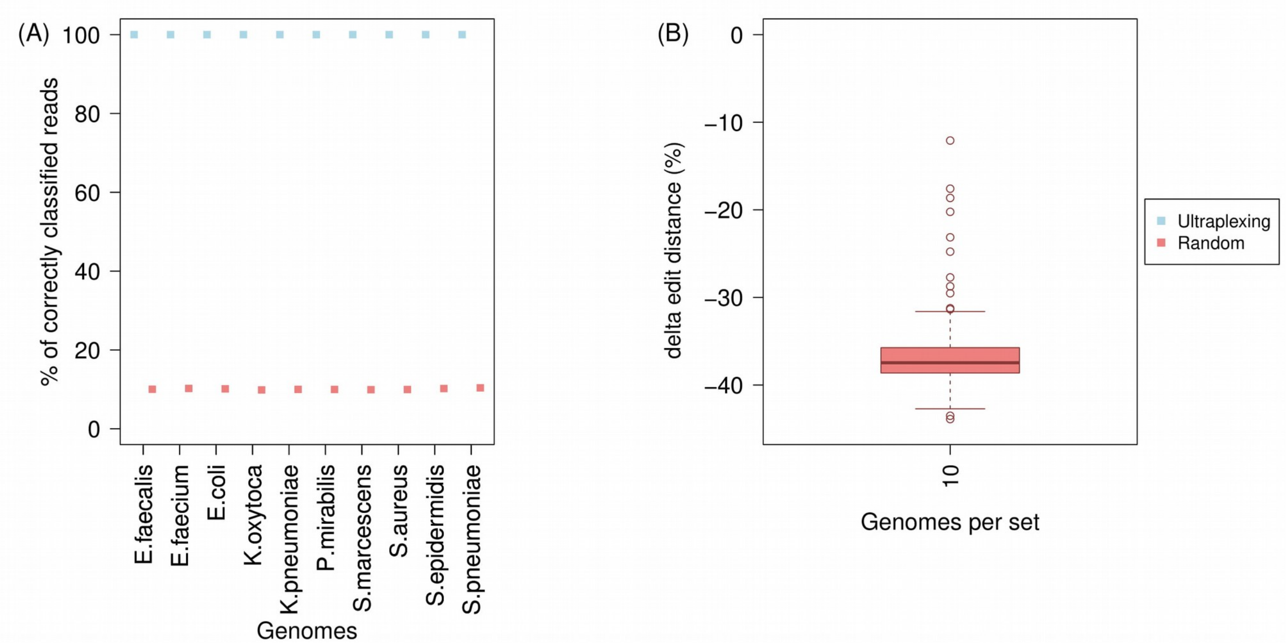

Figure S1: Read classification in a simulation experiment with ten different human pathogens. The figure shows the percentage of correctly classified simulated long reads (A) and Δedit distance for falsely classified reads (B). Reads were assigned according to the Ultraplexing algorithm (Ultraplexing) and randomly (Random).

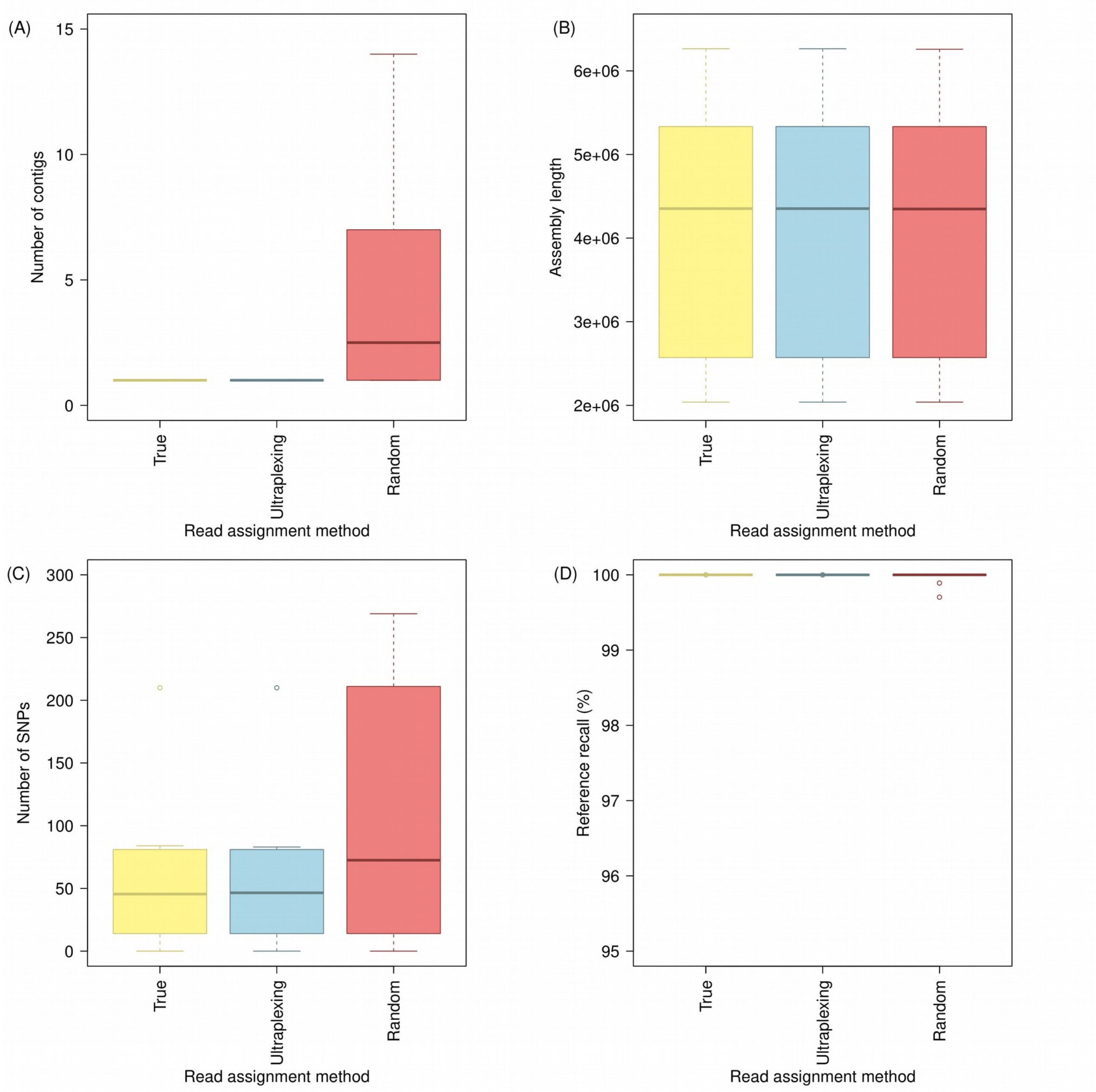

Figure S2: Assembly accuracy in a simulation experiment with ten different human pathogens. The figure shows the distribution of contigs per assembly (A); the distribution of assembly lengths (B); the distribution of SNPs per assembly (C); and the distribution of reference recall (D). Long reads were assigned to their true origin (True); by the Ultraplexing algorithm (Ultraplexing); and randomly (Random). Independent of long-read assignment method, the same simulated short-read data are used for all hybrid assemblies of the same species. SNPs and reference recall were calculated relative to the utilized reference genomes.

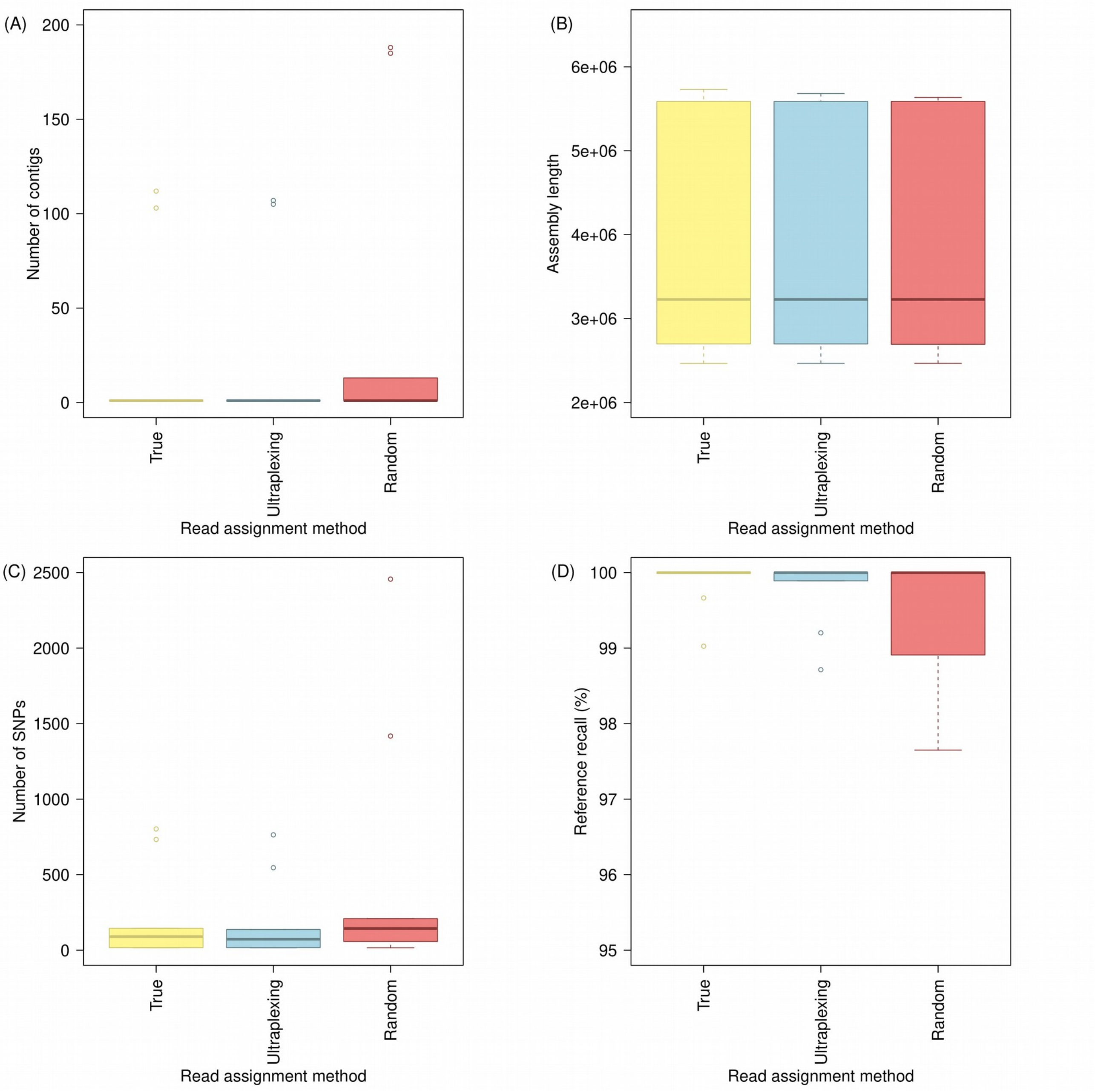

Figure S3: Assembly accuracy in a simulation experiment with five different human pathogens, each represented by two closely related strains. The figure shows the distribution of contigs per assembly (A); the distribution of assembly lengths (B); the distribution of SNPs per assembly (C); and the distribution of reference recall (D). Long reads were assigned to their true origin (True); by the Ultraplexing algorithm (Ultraplexing); and randomly (Random). Independent of long-read assignment method, the same simulated short-read data are used for all hybrid assemblies of the same species. SNPs and reference recall were calculated relative to the utilized reference genomes.

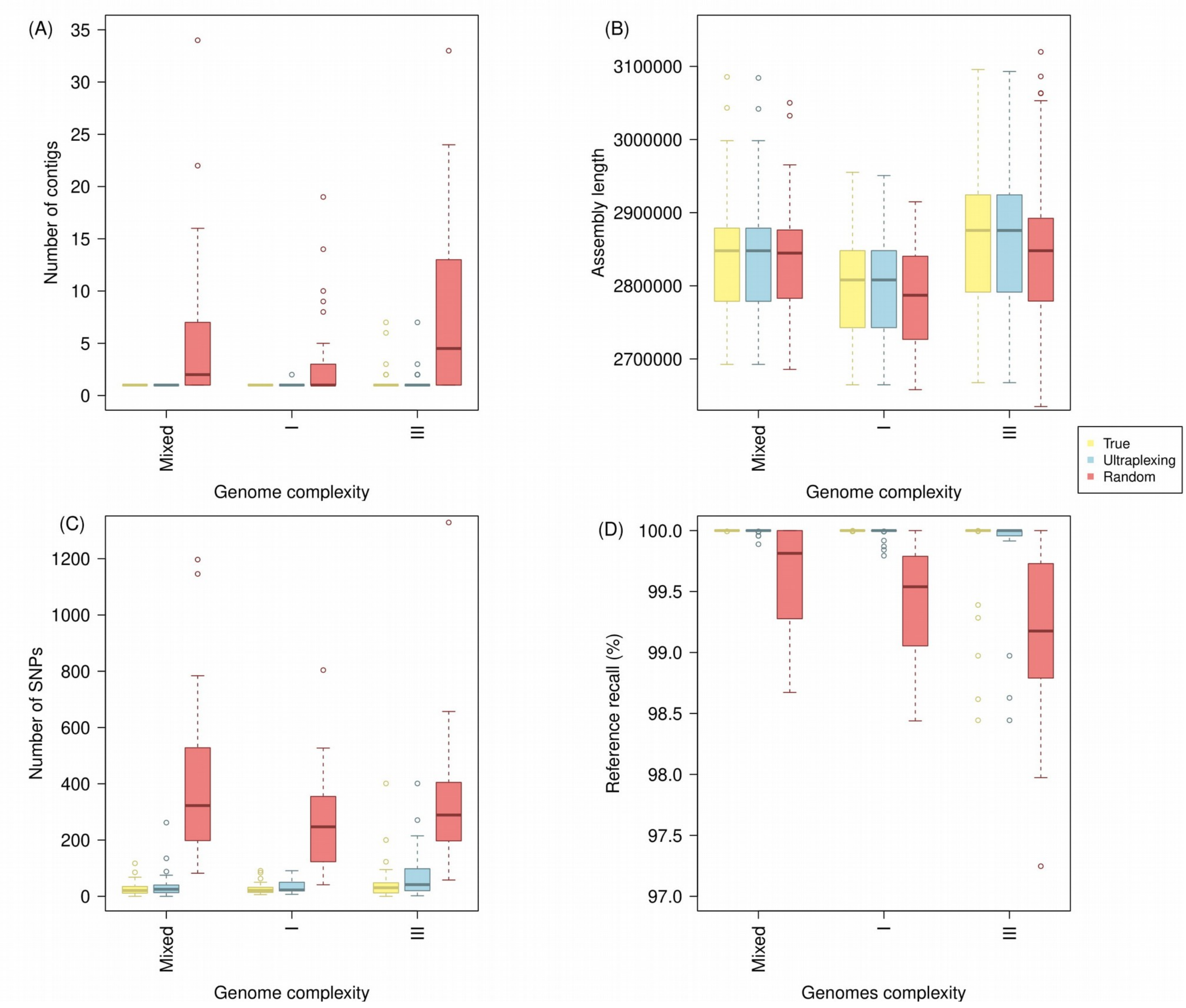

Figure S4: Assembly accuracy in three simulation experiments with 30 S. aureus genomes of different genome complexity each, based on 30 genomes with mixed complexity randomly drawn from the set used for the main part of Simulation experiment II (Mixed); 30 class I complexity (I) genomes; and 30 class III complexity (III) genomes. The figure shows the distribution of contigs per assembly (A); the distribution of assembly lengths (B); the distribution of SNPs per assembly (C); and the distribution of reference recall (D). Long reads were assigned to their true origin (True); by the Ultraplexing algorithm (Ultraplexing); and randomly (Random). Independent of long-read assignment method, the same simulated short-read data are used for all hybrid assemblies of the same isolate. SNPs and reference recall were calculated relative to the utilized reference genomes.

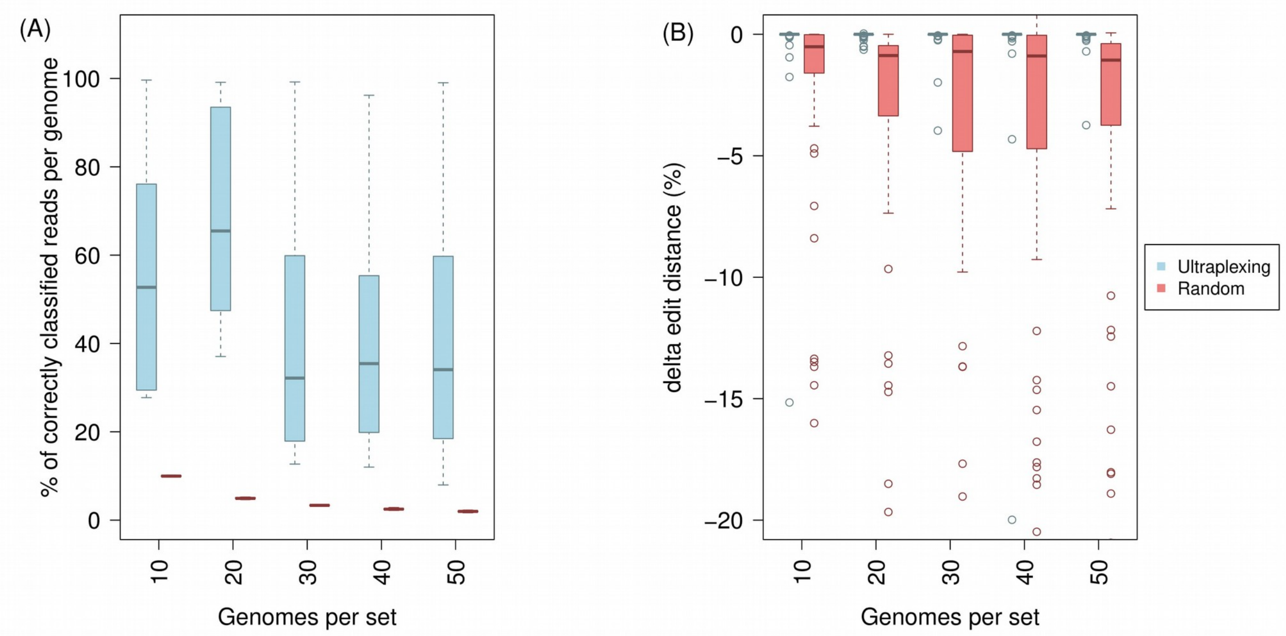

*Figure S5: Read classification in five simulation experiments with 10 – 50 different plasmid-containing S. aureus genomes. The figure shows the distribution of the percentage of correctly classified simulated long reads (A) and the distribution of Δedit distance for falsely classified reads (B). Reads were assigned by the Ultraplexing algorithm (*Ultraplexing*) and randomly (Random).*

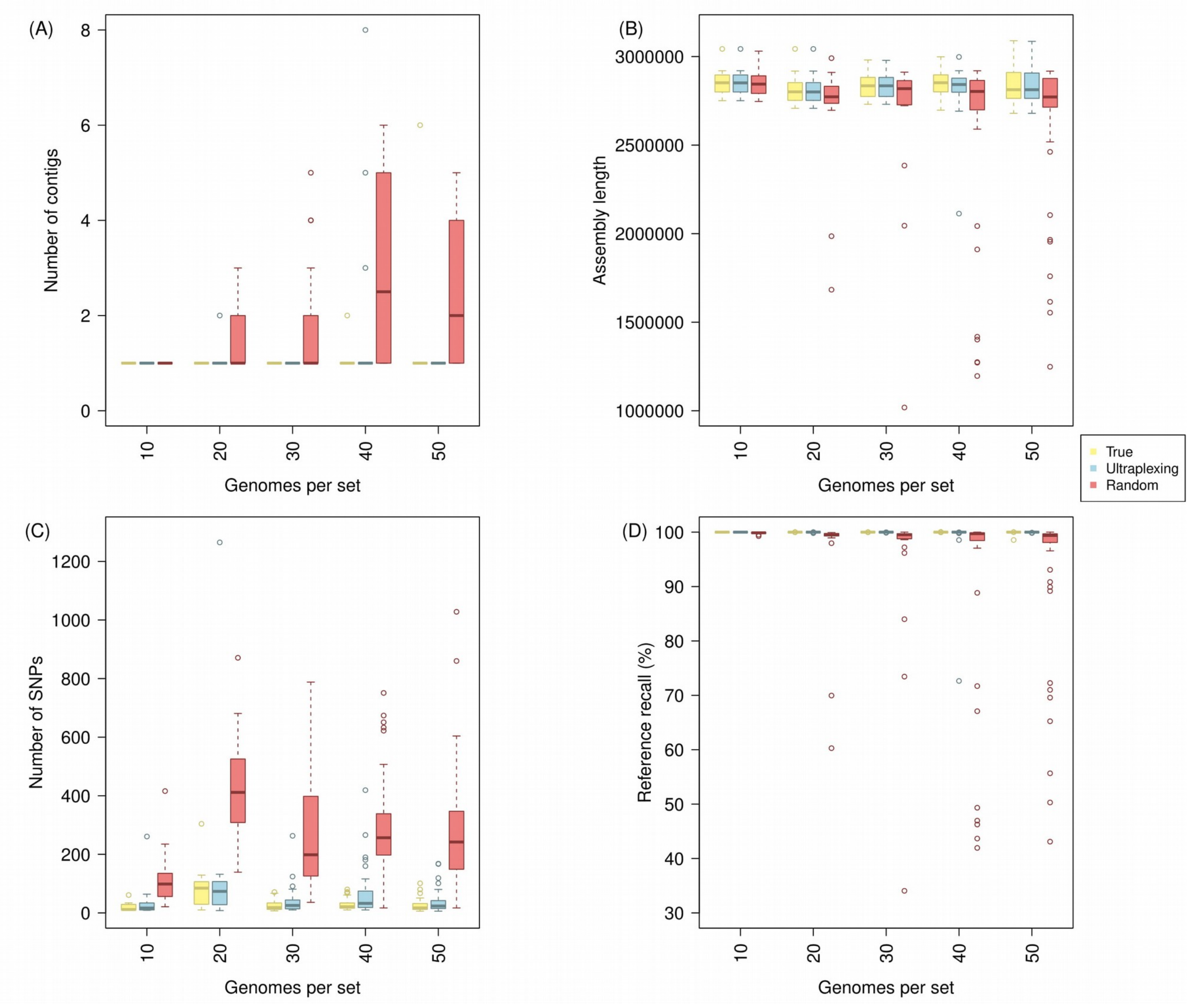

Figure S6: Chromosomal assembly accuracy in five simulation experiments with 10 – 50 different plasmid-containing S. aureus genomes. Reference and assembly contigs were classified as 'chromosomal' or 'plasmid' and evaluated separately (see Methods); shown here are results for the 'chromosomal' compartment. The figure shows the distribution of contigs per assembly (A); the distribution of assembly lengths (B); the distribution of SNPs per assembly (C); and the distribution of reference recall (D). Long reads were assigned to their true origin (True); by the Ultraplexing algorithm (Ultraplexing); and randomly (Random). Independent of long-read assignment method, the same simulated short-read data are used for all hybrid assemblies of the same isolate. SNPs and reference recall were calculated relative to the utilized reference genomes.

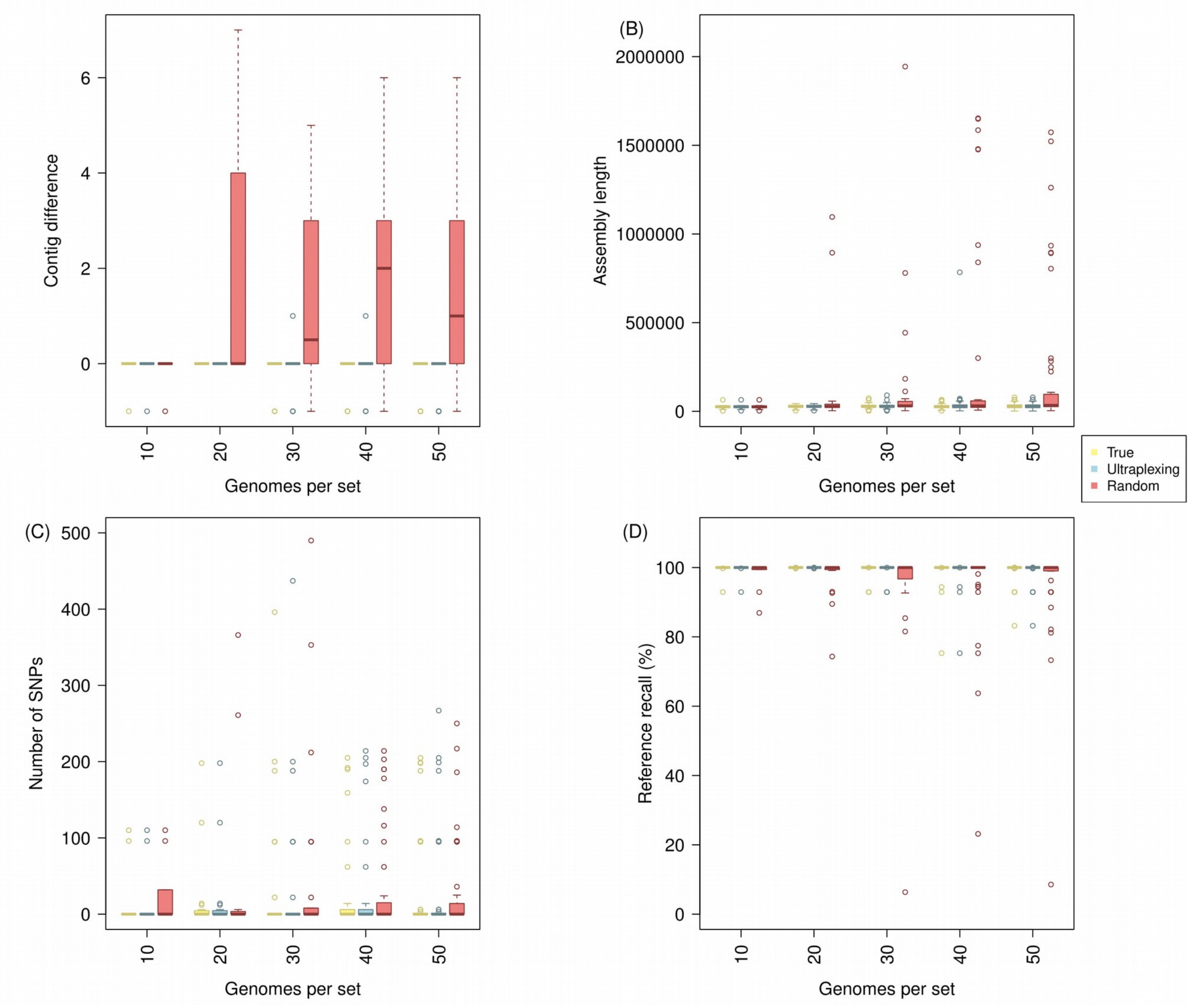

Figure S7: Plasmid assembly accuracy in five simulation experiments with 10 – 50 different plasmid-containing S. aureus genomes. Reference and assembly contigs were classified as 'chromosomal' or 'plasmid' and evaluated separately (see Methods); shown here are results for the 'plasmid' compartment. The figure shows the distribution of contigs per assembly (A); the distribution of assembly lengths (B); the distribution of SNPs per assembly (C); and the distribution of reference recall (D). Long reads were assigned to their true origin (True); by the Ultraplexing algorithm (Ultraplexing); and randomly (Random). Independent of long-read assignment method, the same simulated short-read data are used for all hybrid assemblies of the same isolate. SNPs and reference recall were calculated relative to the utilized reference genomes.

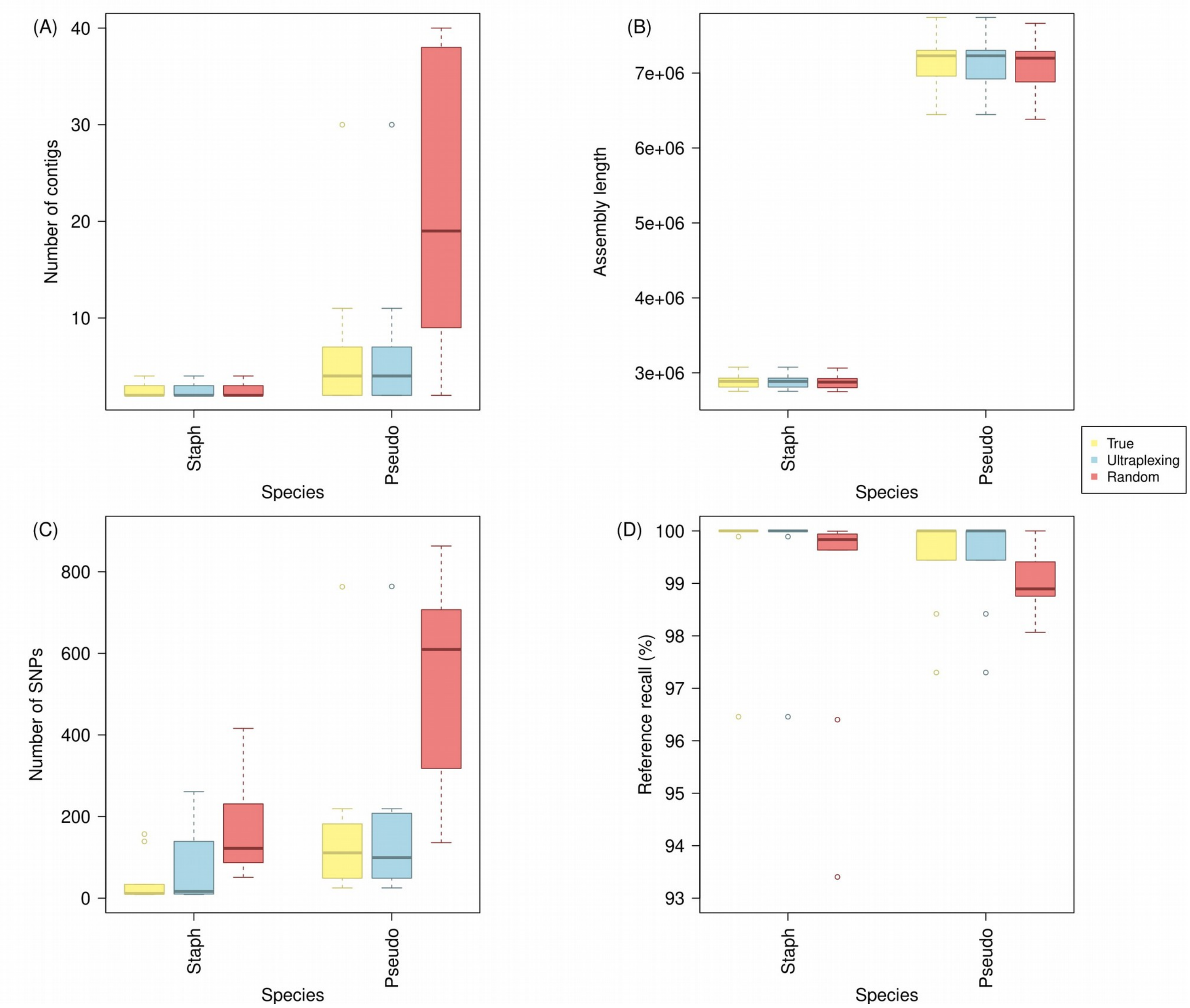

Figure S8: Assembly accuracy in two simulation experiments with 10 plasmid-containing genomes each, based on 10 S. aureus genomes randomly drawn from the set used for the main part of Simulation experiment II (Staph) and 10 Pseudomonas genomes with high repeat richness (Pseudo). The figure shows the distribution of contigs per assembly (A); the distribution of assembly lengths (B); the distribution of SNPs per assembly (C); and the distribution of reference recall (D). Long reads were assigned to their true origin (True); by the Ultraplexing algorithm (Ultraplexing); and randomly (Random). Independent of long-read assignment method, the same simulated short-read data are used for all hybrid assemblies of the same isolate. SNPs and reference recall were calculated relative to the utilized reference genomes. Metrics for the S. aureus isolates were calculated for the chromosomal genome as described in the Methods section, metrics for the Pseudomonas isolates for the complete genome, not distinguishing between chromosomal and plasmid contigs.

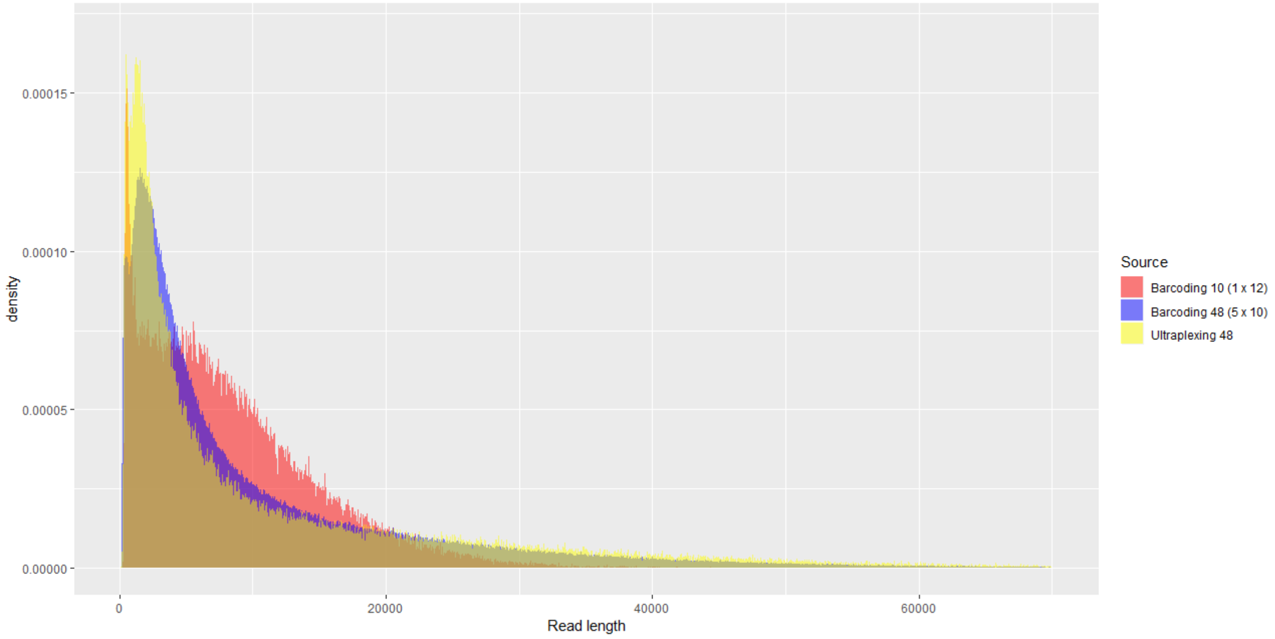

Figure S9: Read length distributions of the generated Oxford Nanopore datasets.
