# Supplementary material for: Ultraplexing: increasing the efficiency of long-read sequencing for hybrid assembly with k-mer-based multiplexing
Source: Genome Biol. 2020 Mar 14;21:68. doi: 10.1186/s13059-020-01974-9 (PMC7071681; doi:10.1186/s13059-020-01974-9)
Supplement: Supplementary file 13 — Additional file 13. Review history. [file 13059_2020_1974_MOESM13_ESM.docx]

**First round**

**Reviewer 1**

The manuscript by Dilthey, Meyer and Kaasch entitled "Increasing the efficiency of long-read sequencing for hybrid assembly with k-mer based multiplexing" describes a novel method termed "Ultraplexing" that aims to improve the de novo assembly of bacterial genomes relying on a combination of long and short read data (a so-called hybrid sequencing approach). Inter-sample genetic variability is used to assign long reads to individual strains of a bacterial mixture. The method aims to overcome the need for barcoding of long read data as generated from platforms like Oxford Nanopore Technologies or Pacific Biosciences.

The manuscript addresses a timely issue of significant interest to the research community. It is concise, clearly written and uses both simulation experiments with the analysis of real datasets to assess the utility and value of Ultraplexing.

I do have a few comments and points that I would like the authors to address to further clarify the relevance of the simulation analyses (considering genome assembly complexity difficulty; see below) and to describe the most relevant use cases for the tool.

#1: The most critical parameter for the simulations and the correct classification of long reads to a certain species/strain is the length of the longest mappable ONT read, which can be significantly shorter than the longest sequenced read. This distinction is not clear from the data presented but needs to be clarified and stated in the Results.

If the longest mappable reads are shorter than the maximal read length parameter that is used, i.e. 61011 (see methods, Simulation experiments), the analysis would have to be repeated. Read size distributions of the mappable reads would be useful additional displays for the real ONT data sets.

Please also indicate whether the longest reads in Table S9 are in fact mappable reads, as this is more relevant. Reports in the literature indicate that the difference between the longest read and the longest mappable read is quite substantial (i.e. the latter being much shorter).

#2. The inter-sample genetic variability is used to assign long reads to individual strains of a bacterial mixture. I wonder whether the selection of bacterial species in simulation experiment #1 is not a bit too simplistic, and whether a mix of 5 clinically relevant species with 2 closely related strains per species could be more relevant to assess the number of simulated long reads that get wrongly assigned to a bacterial strain in the mix of ten strains.

The ten species selected do however capture genomes of different genome assembly complexity (Koren S et al., Reducing assembly complexity of microbial genomes with single-molecule sequencing. Genome Biology 2013); 3 class III genomes (E. faecalis; K. oxytoca; K. penumoniae), 1 class II (E. faecium) and 6 "easy" to assemble class I genomes.

Adding the strain name to the respective Suppl. Tables would be useful in my view.

#3: Simulation experiment 2 considers a very relevant scenario, a mix of 50 strains of one species, in this case Staphylococcus aureus. It would be very relevant for the readership to get an appreciation how such an experiment would turn out for genomes of higher assembly complexity, i.e. going beyond a random sampling but comparing e.g. 30 class I S. aureus genomes and 30 class III S aureus genomes (with can harbor repeats up to 40 kb). If substantially different, such a result could be visualized in Figure 2.

A recent study provided the genome assembly complexity classification for roughly 9600 prokaryotes (Schmid M et al, Pushing the limits of de novo genome assembly for complex prokaryotic genomes harboring very long, near identical repeats. NAR 2018; Table S4), and suitable species and strains of higher assembly complexity could be selected. The Table in that report lists 205 complete S. aureus genomes, 36 of which harbor more complex repeats (calls III genomes).

Overall, the issue of repeat complexity and how it would impact the Ultraplexing results is a relevant point for the readership and should be addressed in the Results/Discussion.

#4: The simulation experiment with the plasmids seemingly would be affected to a greater extent if the selected strains would harbor plasmids that share long repeat sequences with the chromosomes. To explore this more extreme end of the potential influence of plasmids, it could also be carried out on a relevant subset of complex genomes (e.g. P. koreensis described in the paper by Schmid et al mentioned above). This strain harbored several long repeat pairs on chromosome and plasmids.

#5: Are the genome sequences of the ten randomly selected clinical isolates from the real data set deposited in a public repository? Is it known how repeat-rich they are and which genome complexity they would encompass (from class I to III)?

#6: One important point concerns the most relevant applications of Ultraplexing. I agree with the authors that a faster and more cost-efficient characterization of a bacterial mixture is one relevant scenario, e.g. in a diagnostic setting where faster but still highly accurate results can provide significant benefit. In contrast, when aiming to generate high quality reference genome assemblies, the additional time and cost required for a molecular barcoding approach seem well justified to me. I believe this would be a valuable point to be touched upon in the discussion.

#7: The authors did a good job is outlining potential shortcomings in their Discussion. Another point that should be discussed is that the real data only focused on S. aureus, a Gram-positive bacterium for which it might be easier to generate long reads than for Gram-negative bacteria. As mean read length (of mappable reads) will be the critical element for the success of Ultraplexing, this aspect should at least be mentioned. Furthermore, shorter subread distributions of PacBio data will affect the outcome of Ultraplexing (see also point 1); this needs to be clearly stated.

**Reviewer 2**

-Is the Software likely to be of broad utility? Is it easy to install and use?

Although I think that this is likely useful software, I was unable to install it due to insufficient documentation. See below.

-Does the software represent a significant advance over previously published tools (as demonstrated by direct comparison with available related software)?

The software itself seems an advance, but I have described some specific validations regarding runtime below.

-Please indicate briefly the novel features and/or advantages of the software and/or please reference the relevant publications and which alternatives, if any, it should be compared with.

Novel features include the ability to dereplicate pooled nanopore data by comparison to matching Illumina sequencing.

-Are the methods appropriate to the aims of the study, are they well described, and are necessary controls included?

Yes, with a few exceptions described below.

-Are the conclusions adequately supported by the data shown?

There remain some issues, especially relating to Figure 4. See details below.

-Are sufficient details provided to allow replication and comparison with related analyses that may have been performed?

Yes

-Is the paper of broad interest to others in the field, or of outstanding interest to a broad audience of biologists?

Yes, once some qualifications are addressed.

In this paper, Dilthey et al. describe their Ultraplexing approach to simplified hybrid assembly of microbial genomes. Unlike the current state of the art that requires molecular barcodes, the Ultraplexing approach computationally dereplicates pooled Oxford Nanopore reads run together on a single flowcell by aligning them to a de Bruijn graph created using paired Illumina sequencing data from each strain. The authors present several experiments to show that this approach can generate highly accurate assemblies, and suggest that its avoidance of complicated library preparation protocols makes it superior to the current state of the art. Overall, I thought that the author's results were quite convincing and that the approach has merit. However, there are places where I felt that the paper required further clarification.

Major comments:

(1)	The authors make their UltraPlexer software available via a github repository, which I was able to successfully access. However, further details are needed to describe the full installation procedure. In particular, the operating systems on which the software has been tested were not stated, and the list of prerequisites is incomplete (e.g., required Perl modules). As a result, I was unable to install the software in the ~1 hr that I had available (I got stuck trying to convince Math::GPL to install, it seems that it is not natively compatible with the current Ubuntu 18.04 libgsl0-dev libraries). Clarifying and increasing the robustness of the installation procedure will be critical for broad uptake of the Ultraplexing approach.

(2)	In my view, the Read-data experiment II using 48 isolates is problematic. As I understand it, the reference genomes generated from the 48 isolate sequencing data were substandard for some reason, but that these issues weren't present when 10 of these isolates were resequenced. Why the first run produced poor data isn't clear, although the authors speculate that the shorter nanopore reads are to blame (L. 220). However, there are several other potential causes, e.g., bad Illumina or Nanopore reagents, differences in coverage, or just plain old unexplained day-to-day variability. Some of these could be teased apart (e.g., by assembling the Illumina and Nanopore data by themselves and comparing them to the references to see which is the problem), but given that rerunning part of the data fixed the problem I think that the more conservative approach is to write off the 48 isolate data as bad and not consider them further. I don't think that removing Figure 4 and the comparisons to the 48 isolate data in Figure 5 are fatal to the main conclusions of this work, although they do limit the scope of the validation somewhat. Stephen Heard talks about the pitfall of presenting data chronologically instead of as a coherent story in his "The Scientist's Guide to Writing", and this seems exactly the pitfall here.

(3)	Because the Ultraplexing approach relies on Illumina sequencing to generate the underlying de Bruijn graph, it seems that it relies on this being essentially complete. Unfortunately, my group (and others) has shown that this is unlikely to be true for certain bacterial genomes, in particular those with high %GC contents (Goldstein, Beka et al. 2019 BMC Genomics 20:23). This limitation should be included as a caveat of the Ultraplexing approach.

(4)	L. 199 and following states that 48-sample Ultraplexing is more accurate than 24-sample Ultraplexing, but I'm not at all certain of the logic here. Figure 2 seems to argue the opposite. It may be that this conclusion is based on Figures 4 and 5 (it's not clear from the discussion), but as I described above I don't that we can generalize far using those suspect data.

(5)	LL. 209-216: Ultraplexing decreases library prep time by ~9 hours, based on the author's estimates. However, it seems that this gain could easily be swamped by the 70 CPU hours needed for data analysis. Unless the algorithm is multithreaded or run on a cluster it seems that the computational cost outweighs the molecular biology savings. This would contradict the main claim of the paper (that Ultraplexing is more efficient) and so it is critical that full life cycle costs are calculated more clearly.

(6)	More details are needed when describing the Ultraplexing read assignment algorithm. In particular, it isn't currently clear what data (Illumina or MinION) is being assembled by Cortex (I presume Illumina?), and how the output of this step is fed to Unicycler for further assembly. Also, the choice of k=31 seems small for modern Illumina data and should be justified. More generally, is there a cost to using non-optimal reference assemblies for alignment? Why not use something like full SPAdes/Unicycler assemblies for mapping the Nanopore data? I am assuming speed could be an issue, but this isn't specified.

(7)	The UltraPlexer algorithm randomly assigns long reads to a genome when there is an equally confident match between options. I wonder what the consequence of this is compared to discarding such reads instead? Naively, I imagine that removing reads would be beneficial when sequencing a diverse pool of templates (by excluding the reads that are most likely to introduce errors) but not when the pool is highly similar (too many reads would be dropped). The authors might comment on this as part of their discussion of use cases for their method.

(8)	Further details should be provided in the text regarding the composition of the 10 strains used for Simulation experiment I, especially how related these are to each other. This would also clarify the diversity of genomes on which UltraPlexer has been tested (e.g., in regards to size, repetitiveness, and %GC).

(9)	Simulated long-read data was generated using pbsim, but this is designed to simulate PacBio data, not Nanopore. Justification of this (e.g., by explaining the parameter choices used) seems important given that this paper focuses on Nanopore data.

Minor comments:

(10)	LL. 34-35: please clarify what you mean here by "technical microbiology"

(11)	Throughout: replace "MinIon" with "MinION"

(12)	L. 57: Oxford Nanopore now has a 96 barcode kit available (EXP-PBC096), making this new maximum number of samples that can be combined in a run.

(13)	LL. 190-191: "Illumina sequencing enables the reliable characterization of sample genome structure at the level of k-mers." - This seems an odd statement to me, why involve k-mers here? It seems more direct to say something like "Illumina sequencing is more accurate than Nanopore" if this is what you mean.

(14)	LL. 204-206: errors seem to occur in all of the hybrid assemblies. It may be that this is a consequence of any sort of hybrid assembly, e.g., errors introduced by Unicycler. This possibility should be mentioned.

(15)	LL. 235-238: what exactly do you mean by your "long-read-first" approach? Aligning mixed Illumina reads to a long-read assembly? What value with this add to the state of the art?

(16)	You might consider if reference 12 is worth citing, given that it was basically a negative evaluation of a very early MinION release. I'm not sure that is usefully bolsters the idea that MinION is a successful technology.

**Response to reviewers**

**Reviewer 1**

#1: The most critical parameter for the simulations and the correct classification of long reads to a certain species/strain is the length of the longest mappable ONT read, which can be significantly shorter than the longest sequenced read. This distinction is not clear from the data presented but needs to be clarified and stated in the Results. If the longest mappable reads are shorter than the maximal read length parameter that is used, i.e. 61011 (see methods, Simulation experiments), the analysis would have to be repeated. Read size distributions of the mappable reads would be useful additional displays for the real ONT data sets. Please also indicate whether the longest reads in Table S9 are in fact mappable reads, as this is more relevant. Reports in the literature indicate that the difference between the longest read and the longest mappable read is quite substantial (i.e. the latter being much shorter).

We thank the reviewer for this comment! We agree that it is important to specifically investigate the properties of mappable reads. The longest mappable read has a read length of 74kb in the first set of real ONT data (barcoded sequencing of 10 isolates); of 182kb in the second set of real ONT data (barcoded sequencing of 48 isolates; 5 separate runs); and of 208kb in the third set of real ONT data (Ultraplexing-based sequencing of 48 isolates). In addition, the mean read length of mappable reads is 8.4kb in the first set of real ONT data; 11.7kb in the second set; and 10.4kb in the third set. The chosen simulation parameters (max. read length 61kb, mean read length 8.4kb) are therefore conservative.

As requested, we now separately report mappable read statistics in Supplementary Table 10, and we have added a Supplementary Figure that shows the read size distributions of the real ONT data sets (Supplementary Figure 9).

#2. The inter-sample genetic variability is used to assign long reads to individual strains of a bacterial mixture. I wonder whether the selection of bacterial species in simulation experiment #1 is not a bit too simplistic, and whether a mix of 5 clinically relevant species with 2 closely related strains per species could be more relevant to assess the number of simulated long reads that get wrongly assigned to a bacterial strain in the mix of ten strains. The ten species selected do however capture genomes of different genome assembly complexity (Koren S et al., Reducing assembly complexity of microbial genomes with single-molecule sequencing. Genome Biology 2013); 3 class III genomes (E. faecalis; K. oxytoca; K. penumoniae), 1 class II (E. faecium) and 6 "easy" to assemble class I genomes. Adding the strain name to the respective Suppl. Tables would be useful in my view.

We thank the reviewer for raising these important points. As suggested, we have added another simulation experiment in which we create a synthetic mixture of 5 clinically important species, each represented by two closely related strains (mash distance < 0.01). The selected species represent different assembly complexities (2 class I species; 1 class II species; 2 class III species). We report the results of this experiment in the main text and in Supplementary Figure 3.

Consistent with other results, Ultraplexing-based assemblies are of very high quality and virtually identical (for example, identical SNP count observed for 6/10 genomes; Supplementary Table 4, Assembly Multy-species_5x2) in terms of their quality metrics to assemblies based on perfect (i.e. truth-based) read assignment. Of note, slightly reduced assembly qualities for the two included *E. coli* strains are observed for both Ultraplexing- and truth-based assemblies, and probably reflect intrinsic assembly difficulty of the chosen genomes (class III). We discuss these results in the Results section.

As suggested, we have added the names of the strains that the simulations are based on the Supplementary Table 1.

#3: Simulation experiment 2 considers a very relevant scenario, a mix of 50 strains of one species, in this case Staphylococcus aureus. It would be very relevant for the readership to get an appreciation how such an experiment would turn out for genomes of higher assembly complexity, i.e. going beyond a random sampling but comparing e.g. 30 class I S. aureus genomes and 30 class III S aureus genomes (with can harbor repeats up to 40 kb). If substantially different, such a result could be visualized in Figure 2. A recent study provided the genome assembly complexity classification for roughly 9600 prokaryotes (Schmid M et al, Pushing the limits of de novo genome assembly for complex prokaryotic genomes harboring very long, near identical repeats. NAR 2018; Table S4), and suitable species and strains of higher assembly complexity could be selected. The Table in that report lists 205 complete S. aureus genomes, 36 of which harbor more complex repeats (calls III genomes). Overall, the issue of repeat complexity and how it would impact the Ultraplexing results is a relevant point for the readership and should be addressed in the Results/Discussion.

We thank the reviewer for these important suggestions! To further investigate the influence of assembly complexity, we have carried out additional simulation experiments to investigate the performance of Ultraplexing on synthetic mixtures of genomes of different assembly complexity. Specifically, we have assembled sets of 30 difficult (class III) and 30 easy (class I) *S. aureus* genomes, and we compare the accuracy achieved on these to that on 30 arbitrarily selected *S. aureus* genomes from our original selection. Results are presented in the Results section and in Supplementary Figure 4. Assembly accuracy remains very high even for class III genomes (27/30 of assemblies with reference recall and assembly precision >99.9 for both Ultraplexing and perfect read assignment), and in many cases we observe exactly identical assembly accuracy metrics for Ultraplexing- and truth-based assemblies (e.g., identical SNP count for 18/30 class III assemblies; Supplementary Table 4). These results are discussed under the “Simulation experiment II” heading in the Results section. We also summarize these results in the Discussion: “**Fourth, while we have shown that Ultraplexing is generally robust against the presence of complex repeat structures, assembly accuracy was slightly reduced for class III genomes.”**

In response to the next point, we have also added simulation experiments involving another species (see below).

#4: The simulation experiment with the plasmids seemingly would be affected to a greater extent if the selected strains would harbor plasmids that share long repeat sequences with the chromosomes. To explore this more extreme end of the potential influence of plasmids, it could also be carried out on a relevant subset of complex genomes (e.g. P. koreensis described in the paper by Schmid et al mentioned above). This strain harbored several long repeat pairs on chromosome and plasmids.

We thank the reviewer for this idea! As suggested, we have included a simulation experiment with 10 *Pseudomonas* species genomes that (all but one) share repeats >500bp with their plasmids, including the *P. koreensis* genome mentioned by the reviewer. As a comparison, we use the set of 10 plasmid-containing *S. aureus* genomes from the other plasmid-specific simulation experiment. Results from this experiment are shown in Supplementary Figure 4 “Assembly Singlespecies_ repeat_richness”. Measured Pseudomonas assembly accuracy remains high at slightly reduced levels (reference recall >97% and assembly precision >99% for 10/10 Pseudomonas isolates). Ultraplexing- and truth-based assemblies are almost identical (reference recall identical for 10/10 isolates; assembly precision, for 9/10; SNPs, identical for 5/10 isolates and absolute difference varying between +23 and -26 per genome). Observed differences in this experiment are therefore mainly due to the intrinsic assembly complexity of the selected genomes, and not caused by the Ultraplexing algorithm. These results are discussed under the “Simulation experiment III” heading of the Results section.

#5: Are the genome sequences of the ten randomly selected clinical isolates from the real data set deposited in a public repository? Is it known how repeat-rich they are and which genome complexity they would encompass (from class I to III)?

We thank the reviewer for pointing out that the assembly sequences were not fully available! The barcoding-based assemblies used as reference genomes have now been uploaded to GenBank (all associated with BioProject PRJNA528186), and all other assemblies (i.e. all assemblies based on Ultraplexing or on random read assignment) are now publicly available via an Open Science Foundation (OSF) repository (http://dx.doi.org/10.17605/OSF.IO/4M9VH).

As suggested, we have added repeat and complexity statistics for all genomes to the supplementary data (Supplementary Table 1). The select ten clinical isolates comprise genomes with complexities ranging from I to III.

#6: One important point concerns the most relevant applications of Ultraplexing. I agree with the authors that a faster and more cost-efficient characterization of a bacterial mixture is one relevant scenario, e.g. in a diagnostic setting where faster but still highly accurate results can provide significant benefit. In contrast, when aiming to generate high quality reference genome assemblies, the additional time and cost required for a molecular barcoding approach seem well justified to me. I believe this would be a valuable point to be touched upon in the discussion.

We would like to thank the reviewer for raising this point and we agree that not all applications are equally suitable to the application of Ultraplexing. We now explicitly address the speed / accuracy / cost trade-offs in the Discussion: “**Improvements in hands-on time do therefore not necessarily translate into decreased time-to-result. [..] For these reasons, the method is best suited to applications in which large numbers of genomes need to be resolved to very high, but not perfect, accuracy, and in which turnaround times on the order of 3 – 5 days are acceptable. Examples of this include bacterial genome-wide association studies and retrospective outbreak sequencing. For other applications, such as the generation of a small number of reference-grade assemblies or time-critical diagnostic applications, conventional barcoding approaches may remain preferable.**”

#7: The authors did a good job is outlining potential shortcomings in their Discussion. Another point that should be discussed is that the real data only focused on S. aureus, a Gram-positive bacterium for which it might be easier to generate long reads than for Gram-negative bacteria. As mean read length (of mappable reads) will be the critical element for the success of Ultraplexing, this aspect should at least be mentioned. Furthermore, shorter subread distributions of PacBio data will affect the outcome of Ultraplexing (see also point 1); this needs to be clearly stated.

We thank the reviewer for pointing towards potential differences between Gram-positive and Gramnegative bacteria and their respective suitability for Ultraplexing-based assembly generation. To investigate this question, we examined the read size distribution of an internally generated Acinetobacter Nanopore sequencing run (Gram-negative), as well as read length statistics from a recently published, publicly available Nanopore sequencing dataset covering multiple types of Gram-negative and -positive bacteria (Nicholls et al., GigaScience 2019, https://doi.org/10.1093/gigascience/giz043).

The following figures reproduce part of Table 2 of Nicholls et al. and show the read length histogram of our internal Acinetobacter sequencing run in comparison to one of our Staphylococcus runs (the dotted lines show the median read lengths of the two sets):

The observed read length distributions indicate that Gram-negative, as well as Gram-positive, bacteria enable the extraction of sufficiently long reads for the application of Ultraplexing. The extraction of sufficiently long fragments of DNA is now part of the Discussion section describing potential limitations of Ultraplexing: “**Third, the application of Ultraplexing requires high molecular weight DNA, the extraction of which may be challenging for certain bacterial species.”**

As requested, we also note that the shorter read lengths of PacBio may lead to decreased accuracies: “However, based on prior work demonstrating successful k-mer-based classification of eukaryotic PacBio reads (25), we expect that Ultraplexing could also be applied to PacBio data, **though the shorter subread distribution of the technology may negatively impact accuracy (31).**”

**Reviewer 2**

Major comments:

(1) The authors make their UltraPlexer software available via a github repository, which I was able to successfully access. However, further details are needed to describe the full installation procedure. In particular, the operating systems on which the software has been tested were not stated, and the list of prerequisites is incomplete (e.g., required Perl modules). As a result, I was unable to install the software in the ~1 hr that I had available (I got stuck trying to convince Math::GPL to install, it seems that it is not natively compatible with the current Ubuntu 18.04 libgsl0-dev libraries). Clarifying and increasing the robustness of the installation procedure will be critical for broad uptake of the Ultraplexing approach.

We thank the reviewer for trying to install our software and would like to apologize for the difficulties experienced!

We have clarified and improved the documentation on GitHub; specifically, we state the external dependencies (cortex_var, R, and the List::MoreUtils and Math::GSL::Randist Perl modules) more clearly. We have also added a section in which we list the operation system environments under which the software was tested (currently CentOS Linux release 7.5.1804 and Ubuntu 18.04.3 LTS). As Ubuntu 18.04 was specifically mentioned by the reviewer, we verified that the Ultraplexing algorithm can be successfully installed under this operating system. We managed to install the required Perl modules via apt-get and cpan:

sudo apt-get install liblist-moreutils-perl

sudo apt-get install libgsl-dev

sudo cpan Math::GSL::Randist

To simplify the installation procedure, we now also provide a Docker package that manages the Perl and Cortex dependencies.

(2) In my view, the Read-data experiment II using 48 isolates is problematic. As I understand it, the reference genomes generated from the 48 isolate sequencing data were substandard for some reason, but that these issues weren't present when 10 of these isolates were resequenced. Why the first run produced poor data isn't clear, although the authors speculate that the shorter nanopore reads are to blame (L. 220). However, there are several other potential causes, e.g., bad Illumina or Nanopore reagents, differences in coverage, or just plain old unexplained day-to-day variability. Some of these could be teased apart (e.g., by assembling the Illumina and Nanopore data by themselves and comparing them to the references to see which is the problem), but given that rerunning part of the data fixed the problem I think that the more conservative approach is to write off the 48 isolate data as bad and not consider them further. I don't think that removing Figure 4 and the comparisons to the 48 isolate data in Figure 5 are fatal to the main conclusions of this work, although they do limit the scope of the validation somewhat. Stephen Heard talks about the pitfall of presenting data chronologically instead of as a coherent story in his "The Scientist's Guide to Writing", and this seems exactly the pitfall here.

We agree that the presentation of the results from this experiment was not clear. In the revised version of the paper, we benchmark the 48-sample Ultraplexing run – which, in our opinion, adds value to the paper – against a set of newly generated high-quality reference genomes.

To generate these, we relied on the approach that had worked well for the first 10 clinical samples (Real-data experiment I), i.e. we used the established 12-barcode molecular barcoding kit for Nanopore and sequenced up to 10 samples on a single flow cell (5 new runs in total).

In presenting the results from this experiment, we now focus on the quality of Ultraplexing-based assemblies, instead of trying to evaluate the relative merits of Ultraplexing v/s 24-sample barcoding. The quality of the Ultraplexing-based assemblies remains high even in a 48-sample setup, although it remains slightly below that of the generated reference genomes (which is to be expected, given that the reference assemblies are based on approximately 5x more long-read sequencing data than the Ultraplexing-based assemblies).

(3) Because the Ultraplexing approach relies on Illumina sequencing to generate the underlying de Bruijn graph, it seems that it relies on this being essentially complete. Unfortunately, my group (and others) has shown that this is unlikely to be true for certain bacterial genomes, in particular those with high %GC contents (Goldstein, Beka et al. 2019 BMC Genomics 20:23). This limitation should be included as a caveat of the Ultraplexing approach.

We thank the reviewer for highlighting this important limitation. We have amended the Discussion accordingly: “**Second, Ultraplexing relies on Illumina data for read assignment and hybrid assembly; systematic biases in Illumina sequencing, as observed for certain bacterial genomes with high or low GC content (32), may affect the accuracy of Ultraplexing.**”

(4) L. 199 and following states that 48-sample Ultraplexing is more accurate than 24-sample Ultraplexing, but I'm not at all certain of the logic here. Figure 2 seems to argue the opposite. It may be that this conclusion is based on Figures 4 and 5 (it's not clear from the discussion), but as I described above I don't that we can generalize far using those suspect data.

Again, we would like to apologize for the unclear presentation! The original statement was meant to refer to 24-sample molecular barcoding, but, as pointed out by the reviewer, it is not clear whether the observed differences can be confidently related to the utilized barcoding kit. For the revision, we have removed this statement.

(5) LL. 209-216: Ultraplexing decreases library prep time by ~9 hours, based on the author's estimates. However, it seems that this gain could easily be swamped by the 70 CPU hours needed for data analysis. Unless the algorithm is multithreaded or run on a cluster it seems that the computational cost outweighs the molecular biology savings. This would contradict the main claim of the paper (that Ultraplexing is more efficient) and so it is critical that full life cycle costs are calculated more clearly.

We thank the reviewer for this point. We have clarified that the efficiency gains of Ultraplexing are realized at the levels of reagent costs and hands-on time, but not necessarily at the level of timeto- result. Once started, Ultraplexing does not require further user interaction, i.e. can be run in the background. In time-to-result-critical applications, generation of sufficient amounts of Illumina data may be an additional limiting factor. We have added a paragraph to the Discussion to more explicitly discuss these trade-offs: “**Improvements in hands-on time do therefore not necessarily translate into decreased time-to-result. [..] For these reasons, the method is best suited to applications in which large numbers of genomes need to be resolved to very high, but not perfect, accuracy, and in which turnaround times on the order of 3 – 5 days are acceptable. Examples of this include bacterial genome-wide association studies and retrospective outbreak sequencing. For other applications, such as the generation of a small number of reference-grade assemblies or time-critical diagnostic applications, conventional barcoding approaches may remain preferable.**”

(6) More details are needed when describing the Ultraplexing read assignment algorithm. In particular, it isn't currently clear what data (Illumina or MinION) is being assembled by Cortex (I presume Illumina?), and how the output of this step is fed to Unicycler for further assembly.

We apologize for insufficient clarity. Cortex only operates on Illumina data, and the cleaned Cortex graph is used exclusively for assignment of the long reads. After read assignment (i.e. after each long read has been assigned to one of the samples), the Cortex graph is discarded. Unicycler receives, for each sample, the sample-specific Illumina data and all long reads assigned to the sample.

We now describe the read assignment and Unicycler process more explicity:

“For each sample, a de Bruijn graph (k = 19) is constructed from the sample-specific Illumina **short-read** data and the graph is cleaned (removal of low-coverage supernodes) with Cortex.

[..]

**After the long-read assignment process is complete (i.e. after each long read has been assigned to one of the n isolates), the Cortex graph is discarded for the subsequent assembly steps.**

[..]

**Unicycler (14) was used for all hybrid assembly experiments in this publication. Unicycler receives, for each sample, (I) the sample-specific Illumina reads and (II) the long reads assigned to the sample. Long reads are assigned according to the Ultraplexing long-read assignment algorithm; molecular barcodes; or the underlying ground truth, depending on the evaluation scenario.**”

Also, the choice of k=31 seems small for modern Illumina data and should be justified. More generally, is there a cost to using non-optimal reference assemblies for alignment? Why not use something like full SPAdes/Unicycler assemblies for mapping the Nanopore data? I am assuming speed could be an issue, but this isn't specified.

We would like to apologize for a typo in the Methods section – by default, the algorithm uses k = 19. The choice of k is motivated by a trade-off between the number of isolate-specific k-mers at a given k and the corresponding k-mer survival rate, in particular for the long-read data. At k = 19 and error rates between 7 and 15%, k = 19 corresponds to survival rates between 5 and 25%; k = 19 was chosen a) based on previous work (Koren et al., Nature Biotechnology 2018, https://dx.doi.org/10.1038%2Fnbt.4277) on k-mer-based long-read binning and b) after preliminary simulation experiments indicated that k = 19 would work well in practice.

We have added the following description of the choice of k to the manuscript: “**Of note, the choice of a k is a trade-off between the number of isolate-specific k-mers at a given k and the expected k-mer survival rate in the long-read data, calculated as (1 – e)^k, where e is the long-read sequencing error rate. k = 19 was chosen based on published work (25) on k-merbased binning of long reads and based on preliminary simulation experiments.**”

We agree with the reviewer that it may also be possible to use mapping (of long reads to assemblies only based on short reads) for the read assignment process. However, a mapping-based long-read assignment process would likely be more complicated: it is not clear how to distinguish between assembly errors and true sample-specific polymorphisms at the level of such candidate assemblies; it would be necessary to develop a scoring scheme that can deal with the complexities of long-read alignment, such as soft clipping, split alignments etc.; and such an approach would not use any information from areas of the genome that fail to assemble from short reads alone.

By contrast, the k-mer based scoring system that we use is conceptionally simple and easy to implement; and it uses, to the extent that a genomic region is well-represented in the Illumina data (subject to GC content etc., as pointed out by the reviewer), information from all areas of the genome. More advanced scoring systems are, however, a direction for further research; as we state in the Discussion, we view the exploration of proper long-read-to-assembly-graph mapping approaches as a particularly promising potential direction.

(7) The UltraPlexer algorithm randomly assigns long reads to a genome when there is an equally confident match between options. I wonder what the consequence of this is compared to discarding such reads instead? Naively, I imagine that removing reads would be beneficial when sequencing a diverse pool of templates (by excluding the reads that are most likely to introduce errors) but not when the pool is highly similar (too many reads would be dropped). The authors might comment on this as part of their discussion of use cases for their method.

We thank the reviewer for bringing up this point and we agree with the reviewer’s assessment. When sequencing closely related isolates, discarding ambiguous reads will likely have a negative effect; when sequencing more divergent genomes, discarding ambiguous reads may be beneficial, but the exact effects are difficult to predict. This is because Unicycler combines information from across the pool of long-read input data when resolving the short-read assembly graph, and the signal from mis-assigned reads would have to overwhelm the signal from correctly assigned reads during this process in order to negatively affect assembly accuracy. What is more, as read classification generally becomes easier as diversity increases, reads that remain ambiguous even when classifying against a diverse set of genomes are likely of low quality and may not induce valid paths through the short-read assembly graph to begin with.

As the high-diversity case is well-covered by the presented simulation experiments, we have decided to leave an exploration of the precise effect of discarding ambiguous reads for future work. We have, however, implemented a new algorithm switch --ignoreAmbiguousReads that enables users to experiment with discarding ambiguous reads; this may, for example, have applications in analysing low-coverage long-read datasets, in which the effect of individual misassigned reads may be more pronounced.

(8) Further details should be provided in the text regarding the composition of the 10 strains used for Simulation experiment I, especially how related these are to each other. This would also clarify the diversity of genomes on which UltraPlexer has been tested (e.g., in regards to size, repetitiveness, and %GC).

We thank the reviewer for this suggestion. We have amended Supplementary Table 1 with statistics on genome size, complexity, repetitiveness, and GC content. To address the question about relatedness, we have added a new Supplementary Table with pairwise mash distances of the genomes used in the simulation experiments (Supplementary Table 2). Briefly, Simulation experiment I covers genomes ranging from 32% to 60% in GC content, from complexity I to III, from 2.0 to 6.3Mb in size. We have added these figures to the text. In the original version of the manuscript, pairwise mash distances ranged from 0.02 to 0.20. In the revised version and in response to a comment from Reviewer 1, we have also included a variant of the experiment in which we combine 5 species, each represented by 2 closely related strains (mash distance < 0.01).

(9) Simulated long-read data was generated using pbsim, but this is designed to simulate PacBio data, not Nanopore. Justification of this (e.g., by explaining the parameter choices used) seems important given that this paper focuses on Nanopore data.

We thank the reviewer for this comment and we agree in principle. However, the Nanopore technology and the associated basecallers have been developing very rapidly, and it has been difficult for simulation tools to keep up with these developments.

We have tried to compensate for the use of pbsim with conservative parameter settings: based on our first Nanopore sequencing run, we are using max. read length = 61kb and mean read length = 8.4 for simulations. Observed values in real Nanopore data were 74kb; 182kb; and 208kb for max. read length and 8.4kb; 11.6kb; and 10.4kb for mean read length (see Supplementary Table 10 and our response to reviewer 1). For accuracy, we’ve specified a mean accuracy of 88%. On the one hand, 88% is the current modal accuracy of non-CCS PacBio sequencing; on the other hand, 88% is safely below the >90% modal accuracy achieved in current 9.4.1 Nanopore sequencing runs. We clarify in the Methods section of the manuscript that our parameter choices are based on the first Nanopore sequencing run: “**Mean read length was adjusted to match that of our first Nanopore sequencing run, and maximum read length was set to approximately 85% of that observed on the first run (Supplementary Table 10).”**

Minor comments:

(10) LL. 34-35: please clarify what you mean here by "technical microbiology"

We have changed the term to “industrial microbiology”.

(11) Throughout: replace "MinIon" with "MinION"

We thank the reviewer for this comment. We have replaced “MinIon” with “MinION” in the complete manuscript.

(12) L. 57: Oxford Nanopore now has a 96 barcode kit available (EXP-PBC096), making this new maximum number of samples that can be combined in a run.

We have clarified that the statement refers to PCR-free approaches: “A maximum of 24 samples can currently be multiplexed on an Oxford Nanopore MinION flow cell **using the manufacturer’s kits for ‘native’ (PCR-free) barcoding**”

(13) LL. 190-191: "Illumina sequencing enables the reliable characterization of sample genome structure at the level of k-mers." - This seems an odd statement to me, why involve k-mers here? It seems more direct to say something like "Illumina sequencing is more accurate than Nanopore" if this is what you mean.

This statement referred to the fact the Illumina sequencing enables the reliable determination of which k-mers are present in an individual genome, and that this property is key to the Ultraplexing approach (as the read assignment algorithm operates on k-mers.

To clarify, we have changed the sentence to: “Ultraplexing […] benefits from the fact **that Illumina sequencing enables the reliable characterization of the k-mer spectra of individual genomes.”**

(14) LL. 204-206: errors seem to occur in all of the hybrid assemblies. It may be that this is a consequence of any sort of hybrid assembly, e.g., errors introduced by Unicycler. This possibility should be mentioned. We agree that these may reflect limitations of the hybrid assembly approach. As requested, we have added this possibility to the Discussion: “**remaining errors in the assemblies based on both Ultraplexing and perfect read assignment may represent residual errors introduced by the hybrid assembly approach”**

(15) LL. 235-238: what exactly do you mean by your "long-read-first" approach? Aligning mixed Illumina reads to a long-read assembly? What value with this add to the state of the art?

This statement referred to the possible option of applying a non-hybrid long-read assembler like Canu to the Ultraplexing-assigned long reads; this approach, however, is probably less relevant for bacterial genomes, e.g. due to likely decreased rates of expected plasmid recovery. We have removed the statement from the revised version of the paper.

(16) You might consider if reference 12 is worth citing, given that it was basically a negative evaluation of a very early MinION release. I'm not sure that is usefully bolsters the idea that MinION is a successful technology.

We thank the reviewer for this comment. We have removed the reference

**Second round**

**Reviewer 1**

The authors have done a great job in addressing the reviewer comments and suggestions; through the revision, they have further enhanced the impact of the manuscript.

A substantial amount of additional work was carried out, which demonstrates a broad utility for their Ultraplexing approach also on Gram-negative bacteria.

Major improvements concern the simulations that now include more complex bacterial genomes and for which both repeats on chromosomes and plasmids were considered. Also, the relavant scenario of multiple closely related strains was addressed.

Numerous changes were made in the revision that underline the value of the approach for a number of different scenarios. Limitations were adequately discussed/put in perspective.

I expect this solution to attract widespread interest from the community and to be heavily used.

**Reviewer 2**

I congratulate the authors on an improved manuscript that clarified my issues with the previous version. I can confirm that the software runs, and adding the docker container will certainly help make the program more accessible and useful to the community. I have only a few outstanding editorial comments (I apologize for missing some of these the last time):

(1)	The Koren et al. 2013 Genome Biology reference should be cited at first mention of the Class I and III genomes (L. 134).

(2)	Version numbers are needed for Unicycler, edlib, and BWA-MEM.

(3)	In the QV score calculation, what is averaged for the “average genome size” isn’t explicitly defined. Is this the average of the query and reference genome in the nucmer analysis? I also suggest replacing “QV value” with “QV score”, because if “QV” means “Quality Value” this leads to “Quality Value value” when the abbreviation is expanded.

(4)	References need to be checked carefully. Ref. #3 lacks an article number, several online journals are cited as websites instead of journal articles (identified by their having “[Internet]” and citation dates instead of article numbers), and #35 is incomplete.

(5)	“S.aureus” should be italicized in the legends for Figures 2-4.
